# 400 million voting records show profound racial and geographic disparities in voter turnout in the United States

**DOI:** 10.1371/journal.pone.0268134

**Published:** 2022-06-08

**Authors:** Michael Barber, John B. Holbein

**Affiliations:** 1 Department of Political Science, Brigham Young University, Provo, UT, United States of America; 2 Frank Batten School of Leadership and Public Policy, University of Virginia, Charlottesville, VA, United States of America; Vanderbilt University, UNITED STATES

## Abstract

One of the core tenets of a well-functioning representative democracy is that the people who vote to elect government officials are representative of the public. Here we reinforce the idea that reality is far from this lofty ideal. We document the extent and nature of inequities in voter participation in the United States with a level of granularity and precision that previous research has not afforded. To do so, we use a unique nationwide dataset of approximately 400 million validated voting records across multiple election cycles. With this novel dataset, we document large and persistent gaps in voter turnout by race, age, and political affiliation. Minority citizens, young people, and those who support the Democratic Party are much less likely to vote than whites, older citizens, and Republican Party supporters. Minorities, youth, and democrats are also much more likely to live in local communities where fewer individuals vote—areas that we term *turnout deserts*. Turnout deserts are especially pernicious given that they are self-reinforcing—bolstered by the social dynamics that fundamentally shape citizens’ voting patterns. Our results show just how glaring inequities in political participation are in the US. These patterns threaten the very fabric of our democracy and fundamentally shift the balance of political power in the halls of government towards the interests of whites, older citizens, and republicans. They illustrate that participation in the United States is strikingly unequal—far from the ideals that this country has long aspired to.

## Introduction

Voting is one of the central pillars of representative government [1]. Indeed—given its core role of shaping who gets elected to positions of power and the public policies they make [2–4]—voting can be thought of as *the* foundational act of democracy [2, 3, 5] and a key component in determining the individual well-being of the multitudes of citizens affected by the policies that government implements. Voting serves as the primary means of ensuring a “government of, by and for the people” [6]. For these reasons, social scientists, philosophers, historians, pundits, and theorists have long looked to voter turnout as a barometer of democracy’s health and have lamented stagnating or declining levels of democratic participation [2, 7, 8]. Advocates of higher turnout have made many attempts to increase turnout, including proposing numerous electoral reforms and spending millions of dollars (and countless hours) each election cycle in an attempt to get-out-the-vote (GOTV) [9].

Despite the critical importance of voting, much of what we understand about this fundamental form of civic participation comes from data sources that have distinct limitations. A dominant majority of voter turnout studies use survey data—either from government sources (e.g. the Current Population Survey [CPS]) or those collected by social scientists (e.g. the American National Election Survey [ANES] or the Cooperative Congressional Election Study [CCES]) [10]. While we have, undoubtedly, learned many things from these studies, this reliance on surveys is unfortunate for at least two reasons. First, most surveys measure *self-reported* levels of voter turnout and, as such, are susceptible to social desirability biases [11]. As we show below, over-reporting of voting is *not* random, with certain groups much more likely to over-report than others. Furthermore, surveys that use validated vote are in reality simply using the *sample* of the voter file that matches to the self-selected respondents to the survey, whereas we use the *entire* file of validated voting individuals, as discussed below.

Second, surveys are rarely large enough to be representative at the local level. Most surveys—including the comparatively large CPS, CCES, and ANES—that contain voting information are only designed to be representative at the state level—not allowing us to dig into the key dynamics at play in local communities [12, 13]. Given this, surveys can say little about the dynamics and consequences of local communities’ patterns of voter participation, and there are therefore significant gaps in what we know about this central act of democracy. We have too little information about who is more/less likely to vote, how voter turnout varies across local communities, and what consequences low/unequal turnout in one’s social network has on future levels of political participation. And much of what we think we know is, in fact, based on potentially biased samples [14].

## Materials and methods

We address these gaps by bringing together a nationwide list of approximately 400 million voter records across two election cycles—2014 and 2016. We focus on these two elections to gather insights from both a Midterm and a Presidential Election and given their proximity to the voter file snapshot that we employ. In the United States, whether a citizen votes (but not who they vote for) is public record. Individual states publish this information. The voter file data that we use in this manuscript has been collated by the data and analytics firm The Data Trust LLC. Much like other large-scale voter-file vendors—like Catalist, L2, and Aristotle—The Data Trust appends voting information from all 50 US states and the District of Columbia. However, unlike other firms that share 1% samples of their voter files with researchers, we have access to the *entire* Data Trust file, which contains just over 200 million individuals in a single year snapshot. Section 1 in S1 File further discusses the benefits of using voter files over survey data to study this question. To explore the extent to which the patterns that we document below persist across electoral contexts, we leverage two nationwide snapshots—one from 2014 (a midterm election) and one from 2016 (a presidential election). Our appended dataset contains voting and registration histories of all registered voters in the United States across these two election cycles. The file contains variables like vote history, age, gender, race, geographic location, and political party (along with a host of other modeled variables). These measures are of high quality. Indeed, [12, 15, 16] show that nationwide files have a high degree of fidelity to historical and contemporary aggregate demographic, partisanship, and turnout measures available through other administrative units (e.g. the Census) as well as individual-level measures in surveys [17]. In some states, race/political party are modeled and in others they are self-reported by the voter—via the voter registration form. The self-reported include AL, FL, GA, LA, NC, SC, MS, TN. The self-reported party states include AK, AZ, CA, CO, DE, DC, MD, MS, NE, NH, NY, NC, OK, OR, RI, UT, WV, and WY. The modeled states use a combination of factors to model race/party, including names, geographic location, previous voting history in primary elections, and campaign data on voter contacts. We show in the S1 File that the patterns that we document of geographic and demographic inequities in voter turnout are not driven by whether a person lives in a modeled or self-reported race/party state. (See discussion in S1 File and S1-S3 and S5-S8 Figs in S1 File for further evidence of the voter file’s high quality).

We focus on calculating three quantities in this paper. First, we estimate the size of turnout gaps by race, age, and political party. When looking at turnout differences by age, we define “old” as greater than 60 and “young” as younger than 30 throughout the paper. Though this quantity has been calculated in survey data (with self-reported turnout) before, it has (to our knowledge) yet to be provided using a comprehensive nationwide administrative file that measure validated voting histories. This is unfortunate as survey-data may provide us with misleading estimates about how large/small these turnout gaps are given differential rates of misreported voter turnout rates. Still, acknowledging that there is a literature on this topic, we readily note that this is not the main contribution of our article. We estimate this quantity first—as a means of bench-marking our results to what has been done previously in the literature. We focus on race, age, and political party given the core role that these factors play in politics in the United States. These estimates provide us with a picture of gaps in political participation across several salient social dimensions.

Second, we identify geographic areas where voter turnout is high and where, in contrast, it is low. We explore the location and types of people who live in what we term a *turnout desert*—a local community where comparatively few individuals around them vote. In this core part of our analysis, we focus our attention on the electoral precinct. We choose precincts because they serve as the geographic unit where most political activity occurs. In the US, precincts vary in size from about 400–3,000 voters [18]. We borrow our terminology here from the public health literature that explores ‘food deserts’ (e.g. [19]) or geographic areas where residents’ access to affordable, healthy food options (especially fresh fruits and vegetables) is restricted. Like a food desert, a turnout desert is a place not necessarily where no one votes, but rather where access to individuals who regularly participate in politics is restricted. Conscientious of the fact that identifying turnout deserts is, in and of itself, a new enterprise, in our analyses we define turnout deserts in different ways—some that rely on where a community lies in the overall distribution of voter turnout, some that rely on arbitrary thresholds of where turnout is low, and some that simply look along the continuous range of turnout levels across communities. (Ultimately, our results are robust to alternative ways of coding turnout deserts.) In contrast to the literature that estimates gaps in voter turnout by citizen demographics, to date no work has identified the scope, prevalence, location, characteristics, and consequences of turnout deserts. This has simply not been possible with survey data that is not large enough nor designed to be representative at levels of geography lower than the state. Simply put, voter files are the only convincing way to look at turnout rates overall and across individual-level subgroups at the micro level. And this has not been done before. This is unfortunate as there are large inequalities in democratic participation within local communities in the United States—a fact that previous research has not fully explored or acknowledged.

Third, we are the first to explore the extent to which registered citizens of various social characteristics are likely to live in turnout deserts. We specifically explore whether minorities, the young, and democrats are more/less likely to live in a turnout desert than whites, older citizens, and republicans. We focus on these dimensions given their salience in struggles for political power in the United States. Again, this task has never been done before given that the data demands have simply been too great. Exploring the extent, nature, and spread of turnout deserts is vitally important given that previous research has suggested that, at its roots, voting is a social act. Indeed, survey-based studies show that individuals that self-report being asked to vote are also much more likely to say that they vote in elections [20, 21], voting interventions that increase the social salience of voting have large effects [22, 23], voting interventions have spillover effects within families [24–26], and individuals with social skills are much more likely to cast a vote [8]. Taken together, this suggests that if certain groups of people—e.g., minorities, Democrats, and youth—live in turnout deserts, these deserts have the potential to be reinforcing—locking-in long-term power inequities in the American political system.

Together, these three quantities serve to give a thorough description of participatory inequalities in American democracy. They provide us with a even clearer picture of turnout inequities than previous research has afforded.

## Results

**Fig 1:**
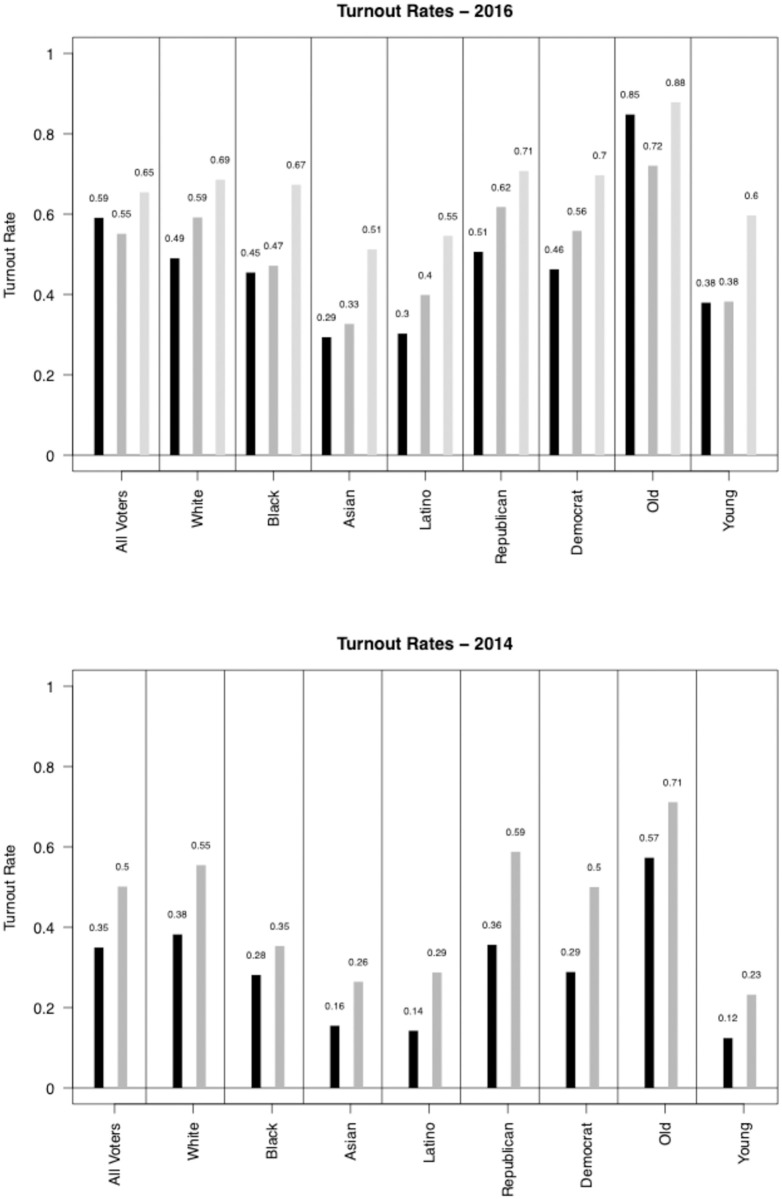
Gaps in validated voter turnout rates by groups using VEP as denominator. Mean levels of voter turnout in 2016 (top) and 2014 (bottom) among individuals in the nationwide voter file (black bars). We use the voting eligible population (VEP) from the US Census for the denominator to determine voting rates. We also report turnout rates using the CCES validated vote (dark grey bars) and the 2016 ANES survey (light grey bars), both of which dramatically over-report turnout in nearly all cases. In all cases “old” is defined as >60 and “young” is defined as <30 years old.

First, we note that there are large gaps across groups in both electoral contexts in addition to major over-estimates of turnout when using survey data (even with validated votes). Fig 1 shows differences by race, party, and age. The black bars are turnout rates using the full voter file. The grey bars indicate turnout rates using CCES validated vote and the ANES, respectively. We note that in many cases survey-based estimates of turnout are much higher than what is found in voter files, which further motivates our recommendation that researchers base turnout rates on voter files and not survey data, even when validated turnout is linked to survey data [12]. (For state by state turnout rates, see S5-S8 Figs in S1 File and for the distribution of turnout in local areas, see S9 Fig in S1 File) Moreover, our work also shows that previous survey measures of turnout are not uniformly higher than voter files; rather, the differences in voter turnout rates vary across individual groups. For example, in 2016 the ANES does a reasonably good job at getting turnout rates for older citizens (i.e. those 60+)—being only 3 percentage points higher than the Data Trust estimate—but the ANES does horribly in getting the turnout rates of younger voters—who they project a full 22 percentage points higher than the Data Trust estimate. Conversely, the CCES validated vote estimates underestimates older citizens’ overall rates of turnout, but gets the overall rate of turnout among young people spot on. (We note, however, that as Fraga and Holbein (2020) show, the CCES’s youth turnout estimates are quite off at the state level.) Similar, although slightly less striking differences can be seen along racial (where misses in the CCES range from 2 to 10 percentage points and 20 to 25 percentage points in the ANES) and partisan (where misses in the CCES range from 10 to 11 percentage points and 20 to 24 percentage points in the ANES) lines. In 2014, racial gaps between the CCES and the Data Trust data range from 7 to 17 percentage points; partisan gaps range from 21 to 23 percentage points; and age gaps range from 11 to 14 percentage points. In short, there is at least some evidence that the difference between voter file estimates of turnout and survey-based (or survey linked to voter files) is not uniform across groups. We note that the differences are not due to social desirability, in the classic sense; that is, in the over-reporting of voting. This is true as we are using the CCES validated voter turnout measures. The differences, then, can be attributed to perhaps the sampling framework of the CCES, those who respond to the survey, or its weights.

Using the rates derived from the voter file (black bars), in 2016 Whites voted at a rate 4 percentage points higher (9% higher relative than the base rate) than Black citizens, 20 percentage points (69%) higher than Asians, and 19 percentage points (63%) higher than Hispanics; Republicans voted at a rate 5 percentage points higher (11%) than democrats; and older citizens (>60 years old) voted at a rate 47 percentage points higher (124%) than younger citizens (<30 years old). In 2014, these gaps were further magnified—Whites voted at a rate 10 percentage points higher (36% greater) than Black citizens, 22 percentage points higher (138%) than Asians, and 24 percentage points higher (171%) than Hispanics; republicans voted at a rate 7 percentage points higher (24%) than democrats; and older citizens vote at a rate 45 percentage points higher (375%) than younger citizens. These gaps are striking. (S4 Fig in S1 File shows these differences using alternative methods of calculating turnout).

**Fig 2:**
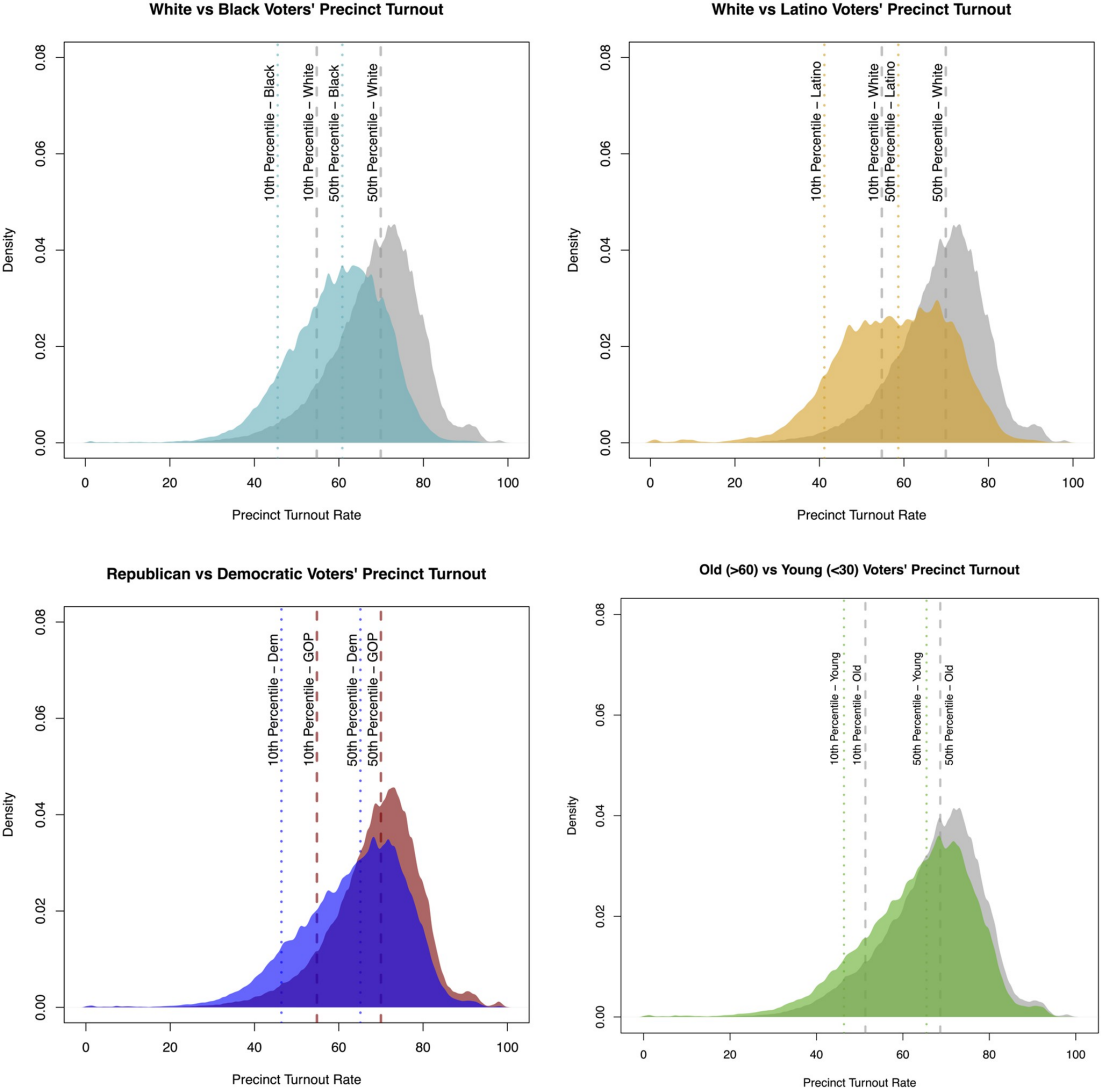
Distributions of validated voter turnout rates by groups distribution of electoral precinct turnout for individuals of various groups in 2016. The first two figures show that Black and Hispanic individuals are much more likely to live in a precinct where few people vote than White citizens (White distribution is colored grey in both figures). The same pattern holds for Democrats (blue distribution) compared to Republicans (red distribution) and to a slightly lesser extent young people (green distribution) compared to older people (grey distribution).

We next transition to our core analyses of voter turnout by social context. We find that the turnout of local communities is highly unequal. This result holds regardless of whether we make arbitrary decisions about what constitutes a turnout desert or, instead, simply look at the continuous distribution of voter turnout across communities. Fig 2 shows the distribution of voting rates in the electoral precincts of voters from several groups. (See also S10 and S11 Figs in S1 File) The top row shows rates of validated voter turnout in Black and Hispanic voters’ precincts (vs. whites) (see S10 and S11 Figs in S1 File for Asians in 2016 and all groups in 2014), while the next two do so for Democrats (vs. Republicans) and younger people’s precincts (vs. older people). As can be seen, there are large gaps along these dimensions. Black, Hispanic, Democrats, and young people are significantly more likely to live in lower turnout areas. This can be seen by the leftward shift of the entire turnout distributions for these groups. In all cases, a Kolmogorov-Smirnov test shows that these distributions are statistically distinct from one another (p <0.0001). Not only are the differences statistically meaningful, they are substantively large. For example, in the second sub-figure (top right) the *median* Hispanic voter’s precinct turnout rate is *barely above the 10th percentile* for whites. The same holds true for Black individuals.

These results show that voter turnout is highly segregated by race, politics, and age in the United States; minorities, young people, and democrats are much more likely to live in turnout deserts. This finding holds if we define a turnout desert based on arbitrary thresholds or cutoffs. For example, if we define a turnout desert as a precinct where turnout was one standard deviation lower than the national average, Black, Hispanic, and Asian individuals are 3, 4, and 2.5 times more likely to live in a turnout desert than whites, respectively. (In the SI we look at alternative definitions of turnout deserts and find similar results.) Likewise, democrats are 2.5 times more likely to live in a turnout desert than republicans. Turnout deserts are also divided by age, albeit less than race and party, perhaps, in part, because age-based segregation is comparatively smaller in the United States. Still, young people are still much more likely (1.6x) to live in a turnout desert than older citizens.

**Fig 3:**
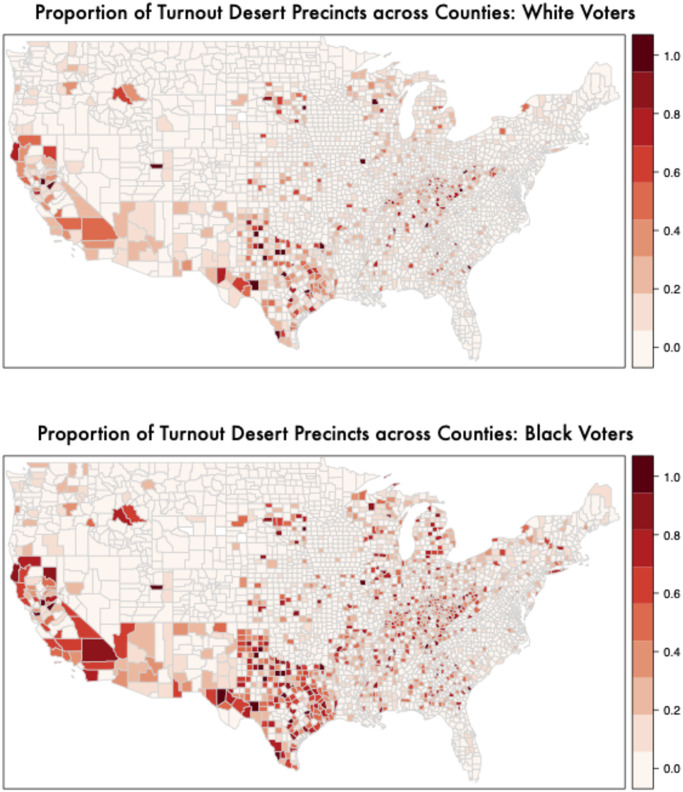
Mapping turnout deserts by racial groups (1) proportion of turnout desert precincts in a county by race (i.e. 1 standard deviation below the national average in voter turnout in 2016). The top panel shows among precincts where White voters live; the second panel shows among precincts where Black voters live. Black (25.8%) individuals are more than 3 times more likely to live in a turnout desert precinct than white voters (8.3%). Minorities’ turnout deserts are spread across large/urban and small/rural locations. S13-S20 Figs in S1 File show similar maps for all groups in 2014 in addition to different definitions of turnout deserts.

**Fig 4:**
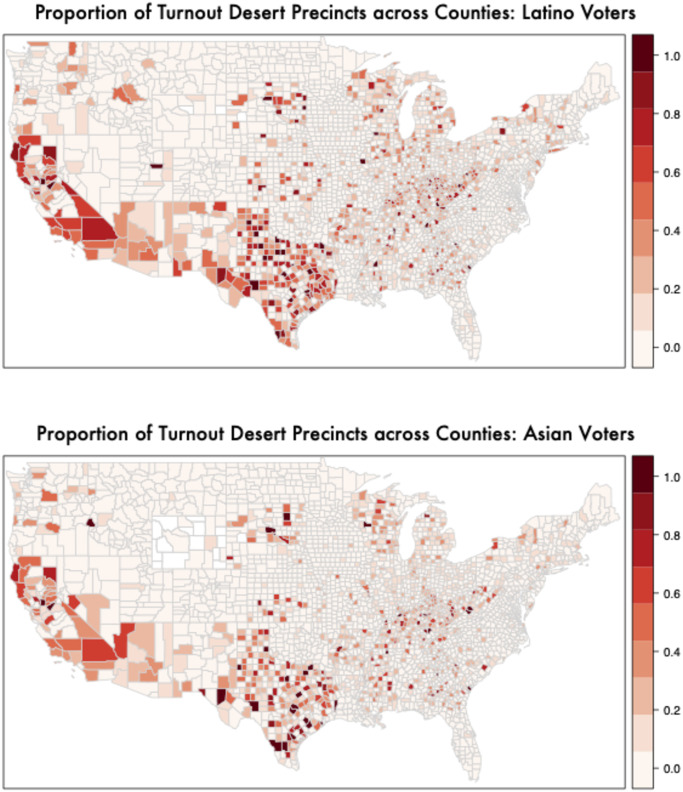
Mapping turnout deserts by racial groups (2) proportion of turnout desert precincts in a county by race (i.e. 1 standard deviation below the national average in voter turnout in 2016). The top panel shows among precincts where Hispanic voters live and the bottom panel shows precincts where Asian voters live. Hispanic (36.2%) individuals are more than 3 times more likely to live in a turnout desert precinct than white voters (8.3%). Asian citizens are 2.6 times more likely to live in a turnout desert precinct than white voters. Minorities’ turnout deserts are spread across large/urban and small/rural locations. S13-S20 Figs in S1 File show similar maps for all groups in 2014 in addition to different definitions of turnout deserts.

Figs 3 and 4 shows the distribution of turnout deserts (precincts where turnout is one standard deviation lower than the national average) across the country, broken down by race by plotting the proportion of precincts in each county that qualify as a turnout desert by this definition. (For other specifications of these maps, see S12-S19 Figs in S1 File) For example, the first panel in Fig 3 considers the precincts where White voters live and calculates the fraction of those precincts (based on total turnout among all voters in those precincts) that are turnout deserts for each county. Similarly, the second panel in Fig 3 considers the precincts where Black voters live and calculates the fraction of those precincts (based on total turnout among all voters in those precincts) that are turnout deserts for each county. It is clear from these figures that minorities are more likely to live in precincts that have overall lower turnout.

In addition to clear demographic patterns (i.e. minorities are more likely to live in areas with very low turnout rates overall) we also see geographic patterns across the country. For example, California, Arizona, and Texas stand out as states with many counties where a large fraction of precincts have remarkably low turnout rates. Counties with high proportions of turnout desert precincts also appear more frequently in the Appalachian region and in the Great Lakes states of Michigan and Wisconsin. However, counties with many precinct turnout deserts appear in both urban and rural parts of country. (S12-S19 Figs in S1 File use alternative definitions of turnout deserts and show that the results presented in the main paper are robust to alternative specifications).

To account for the correlations between race, gender, partisanship and age, we conduct a multiple regression model in which the dependent variable is the turnout rate of each voter’s precinct. We then include each of these demographic variables in the regression model to estimate the independent relationship between the demographic features we have shown above and living in an area with higher or lower turnout. We estimate a separate model for 2014 and 2016 and include state fixed effects in each model. Standard errors are clustered by precinct.

**Fig 5:**
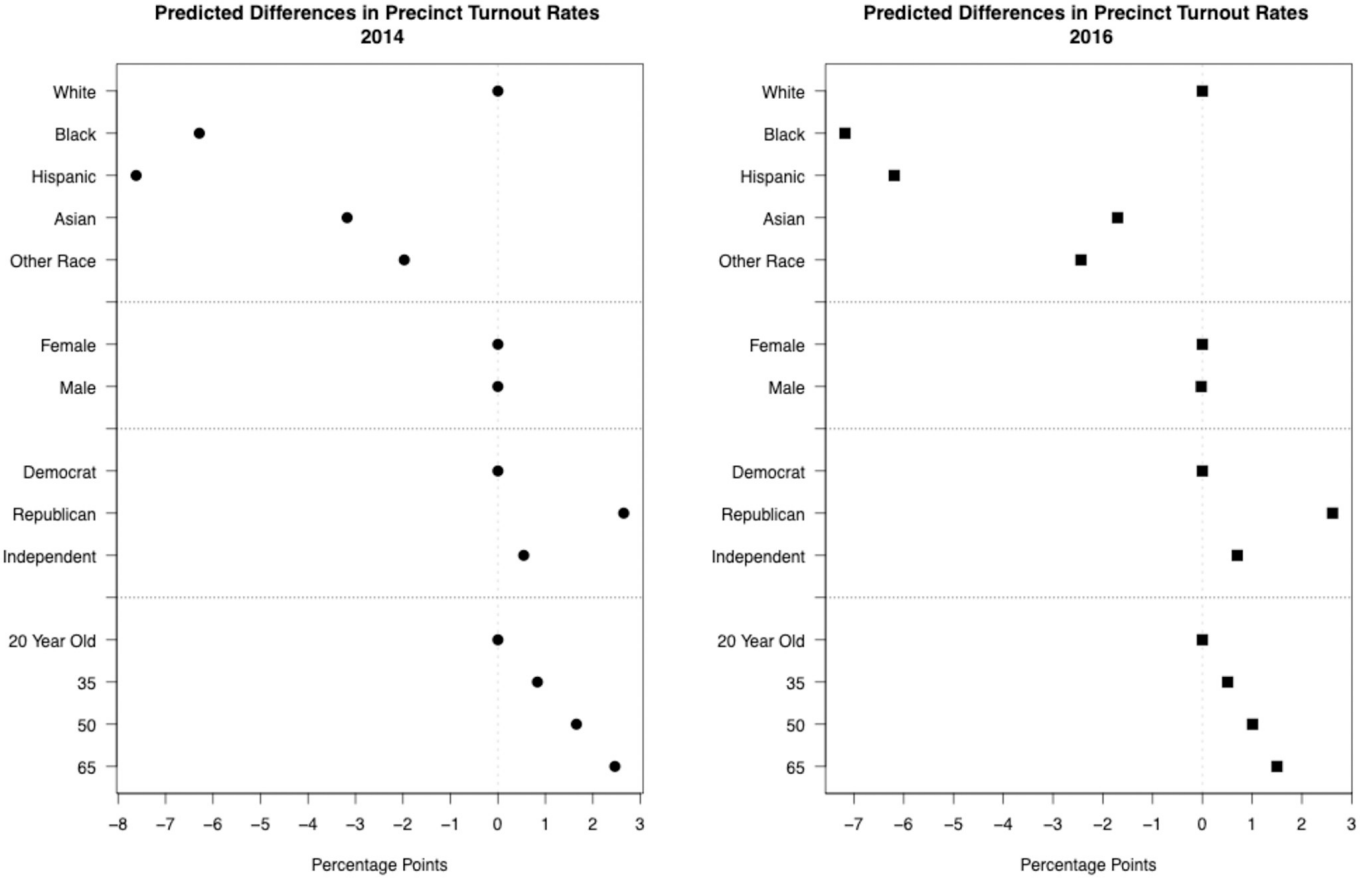
Regression results predicting precinct turnout rate each figure shows coefficients from a model where a voter’s precinct turnout rate is the dependent variable. Coefficients show predicted differences in turnout rates for various demographic characteristics of voters. In both 2014 and 2016 Black and Hispanic voters lived in precincts where turnout was between 7 and 8 percentage points lower than white voters. In both years Republicans lived in precincts where turnout was approximately 3 percentage points higher than Democratic voters. Each model includes state fixed effects and standard errors are clustered by precinct. Confidence intervals are not shown because the very large sample size (150–200 million in each model) leads to intervals that are incredibly small.

Fig 5 displays the results of these regression models. We see that even after accounting for other demographic factors, race, partisanship, and age are strongly associated with living in a precinct with higher or lower turnout. On average, Black and Hispanic voters live in precincts with between 6 and 7 percentage points lower turnout than do whites (the coefficient for White is 0, indicating that it was held out of the regression as the comparison category). Asians were also more likely to live in precincts with lower turnout, but the magnitude is smaller than for Black and Hispanic voters—between 2 and 3 percentage points across the two different elections considered here.

In S1 File we investigate the mechanism for this result and find that it is largely driven by the racial composition of the precinct. (See S20 Fig in S1 File and surrounding discussion.) In other words, racial minorities are individually slightly less likely to vote than white voters, and given the patterns of racial segregation in the United States, racial minorities are also more likely to live in precincts with other people who share their ethnicity. Thus, when aggregated together this leads to the result that minorities are not only less likely to vote individually, but also more likely to live in precincts that have lower turnout, on average, due to the geographic sorting of individuals into racially homogeneous precincts. While we are not the first to point out that racial segregation exists in the United States (far from it), we are one of the first papers to acknowledge that this racial segregation may be politically reinforcing given the socialized nature of voting.

In both years Republicans lived, on average, in precincts where turnout rates were approximately 2.5 percentage points higher than the precinct turnout rates for Democratic voters. We also see that older people are more likely to live in precincts with higher turnout. For each 15 year difference average precinct turnout increases by approximately half a percentage point. There do not appear to be substantial difference in precinct turnout by gender, which is logical given that gender is a demographic factor on which there is the very little geographic polarization or sorting—unlike race, partisanship, and age.

## Discussion

Though not the only form of political power, voting is vitally important given that this act plays a core role in determining who gets elected to government and the policies that they implement. In this paper, we leverage a massive nationwide voter file to show that political power is far from equally distributed in the United States. Though voting is one of the most studied behaviors in the social sciences—filling the pages of thousands of academic articles and books [10]—our work takes an important step by documenting the extent and nature of political participatory inequality in the United States. Though there has been a general sense that this is the case [2], we expose this inequity and all of its dynamics with greater detail and precision than previous work. Our work confirms that minority citizens are much less likely to vote than White citizens, democrats are much less likely to vote than republicans, and young people are much less likely to vote than their elder counterparts.

Furthermore, a key contribution of our article is that it provides the first evidence of the geographic dispersion of political inequality at such a fine micro level and, in so doing, provides evidence of areas in the United States where turnout is strikingly low and unequal. Given the social component of voting, we have strong reason to suspect that turnout deserts are socially stratified in the United States and are self-reinforcing—thus perpetuating political inequality in the United States.

Our work brings the problem of low and unequal turnout into full focus and illustrates that the United States is far from the lofty ideals that this country has aspired to since it was founded. This has special meaning given the current context of large and growing economic inequality [27]. We show that political power is divided along the same social cleavages that separate the economic ‘haves’ from the ‘have nots.’ Just like the economy [27], power in American democracy is fundamentally driven by race, age, and the interests of those best represented by the Republican Party. Our findings suggest that previously identified economic/social inequalities are likely to persist as long as there is insufficient citizen-based pressure on elected officials (through the primary means of doing so—voting) to implement public policies that would change these patterns.

## Supporting information

S1 File Supporting evidence. (PDF)
