# Correction: 400 million voting records show profound racial and geographic disparities in voter turnout in the United States

**DOI:** 10.1371/journal.pone.0314386

**Published:** 2024-11-20

**Authors:** Michael Barber, John B. Holbein

In Fig 1, there are errors in the values due to a computational error. Please see the correct Fig 1 here.

**Fig 1:**
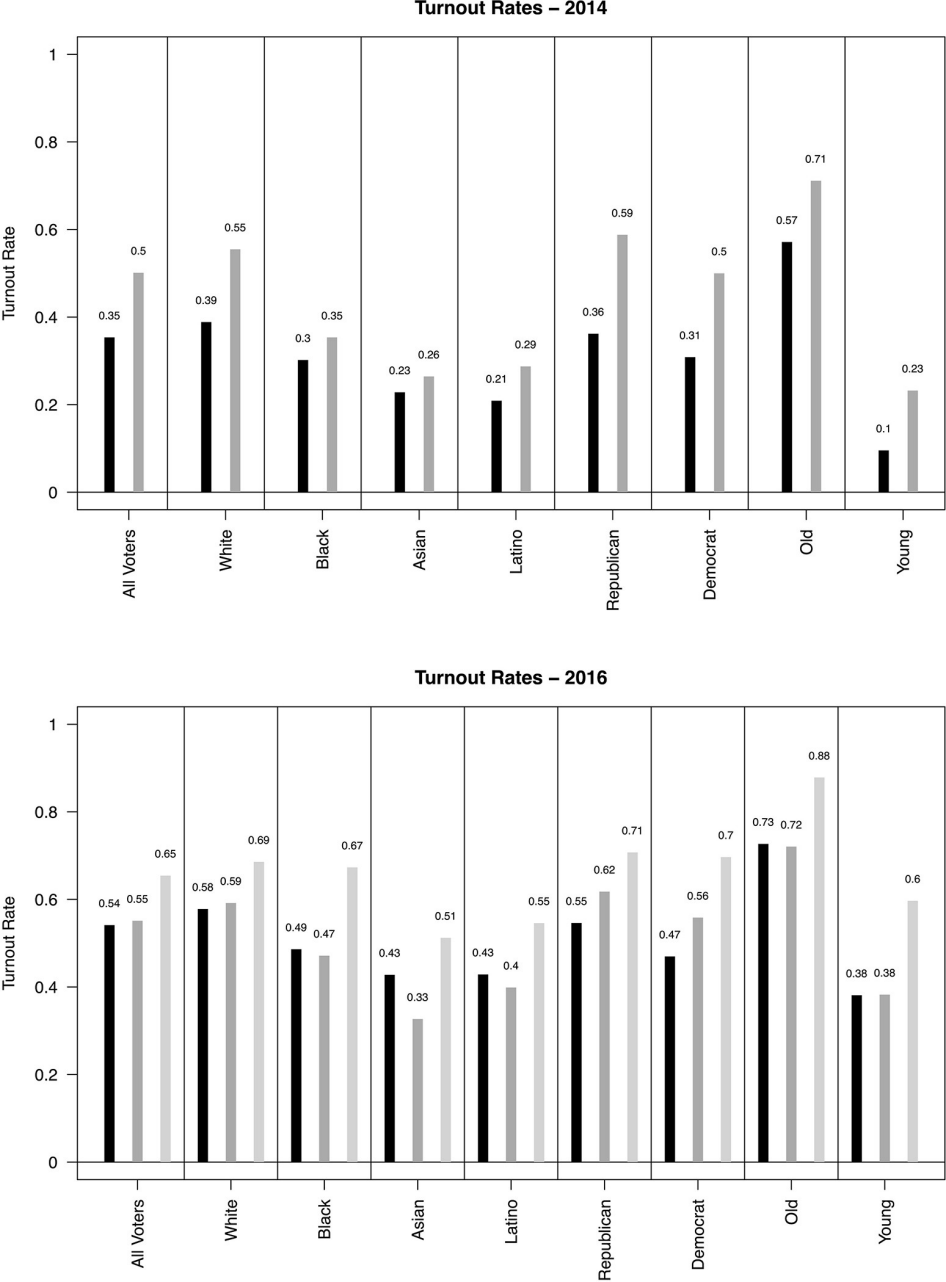
Gaps in validated voter turnout rates by groups using VEP as denominator. Mean levels of voter turnout in 2016 (top) and 2014 (bottom) among individuals in the nationwide voter file (black bars). We use the voting eligible population (VEP) from the US Census for the denominator to determine voting rates. We also report turnout rates using the CCES validated vote (dark grey bars) and the 2016 ANES survey (light grey bars), both of which dramatically over-report turnout in nearly all cases. In all cases “old” is defined as >60 and “young” is defined as <30 years old.

In the Results section, there are errors in the first and second paragraph. The correct paragraph is: First, we note that there are large gaps across groups in both electoral contexts in addition to major over-estimates of turnout when using survey data (even with validated votes). Fig 1 shows differences by race, party, and age. The black bars are turnout rates using the full voter file. The grey bars indicate turnout rates using CCES validated vote and the ANES, respectively. We note that in many cases survey-based estimates of turnout are much higher than what is found in voter files, which further motivates our recommendation that researchers base turnout rates on voter files and not survey data, even when validated turnout is linked to survey data \cite{fraga2019measuring}. (For state by state turnout rates, see S5–S8 Figs in the supplemental materials and for the distribution of turnout in local areas, see S9 Fig.) Moreover, our work also shows that previous survey measures of turnout are not uniformly higher than voter files; rather, the differences in voter turnout rates vary across individual groups. For example, in 2016 the ANES is consistently higher in its estimate of turnout. Conversely, the CCES validated vote estimates come much closer while overestimating and underestimating different subgroups, but gets the overall rate of turnout spot on. (We note, however, that as Fraga and Holbein (2020) show, the CCES's youth turnout estimates are quite off at the state level.) Similar, although slightly less striking differences can be seen along racial (where misses in the CCES range from 1 to 10 percentage points and 10 to 18 percentage points in the ANES) and partisan (where misses in the CCES range from 7 to 9 percentage points and 16 to 23 percentage points in the ANES) lines. In 2014, racial gaps between the CCES and the Data Trust data range from 3 to 16 percentage points; partisan gaps range from 19 to 23 percentage points; and age gaps range from 13 to 14 percentage points. In short, there is at least some evidence that the difference between voter file estimates of turnout and survey- based rates (or survey linked to voter files) is not uniform across groups. We note that the differences are not due to social desirability, in the classic sense; that is, in the over-reporting of voting. This is true as we are using the CCES validated voter turnout measures. The differences, then, can be attributed to perhaps the sampling framework of the CCES, those who respond to the survey, or its weights. Using the rates derived from the voter file (black bars), in 2016 Whites voted at a rate 9 percentage points higher (18% higher relative than the base rate) than Black citizens, 15 percentage points (35%) higher than Asians, and 15 percentage points (35%) higher than Hispanics; Republicans voted at a rate 8 percentage points higher (17%) than Democrats; and older citizens (&gt;60 years old) voted at a rate 35 percentage points higher (92%) than younger citizens (&lt;30 years old). In 2014, these gaps were further magnified—Whites voted at a rate 9 percentage points higher (30% greater) than Black citizens, 16 percentage points higher (70%) than Asians, and 18 percentage points higher (86%) than Hispanics; Republicans voted at a rate 5 percentage points higher (16%) than Democrats; and older citizens vote at a rate 47 percentage points higher (470%) than younger citizens. These gaps are striking. (S4 Fig in the supplemental materials shows these differences using alternative methods of calculating turnout).
