# Supplementary material for: 400 million voting records show profound racial and geographic disparities in voter turnout in the United States
Source: PLoS One. 2022 Jun 8;17(6):e0268134. doi: 10.1371/journal.pone.0268134 (PMC9177044; doi:10.1371/journal.pone.0268134)
Supplement: S1 File — (PDF) [file pone.0268134.s001.pdf]

Online Supplemental Materials:
400 Million Voting Records Show Profound Racial and Geographic Disparities in Voter Turnout in the United States

Michael Barber[a], *John Holbein*[b]

[a]*Department of Political Science, Brigham Young University, Provo, UT*
[b]*Frank Batten School of Leadership and Public Policy, University of Virginia, Charlottesville, VA*

TABLE OF CONTENTS:

This SI includes a number of results noted in the paper, robustness checks and additional models noted in the paper, and additional results not directly mentioned in the paper. Below is a table of contents for this document.

1. Motivating the use of voter files to study individual and geographic patterns of turnout inequality
2. The accuracy of voter file measures
3. Turnout rates for individual states in 2016 and 2014
4. Turnout rates for groups using alternative methods to calculate total number of eligible voters
5. Distributions of precinct turnout rates for Asian voters in 2016 and White, Black, Latino, partisans, old, and young voters in 2014.
6. Maps of Turnout Deserts for Asian voters, Republicans, and Democrats in 2016. Maps of Turnout Deserts for these same groups in 2014.
7. Overview of Methodological Approach.
8. Regression models for Figure 3 in paper with additional model specifications.
9. County-level regression results with citizen voting age population (CVAP) as the denominator.

LIST OF FIGURES

S1 Data Trust Registration Counts and VAP by State . . . .

S2 Data Trust Black Variable and Census Black Population Counts . . . .

S3 Data Trust Modeled Party Relative to Presidential Vote Share (2016) . . . .

S4 Gaps in Validated Voter Turnout Rates by Groups using Voter File as Denominator . . . .

S5 Validated Voter Turnout Rates by State, 2016: Mean levels of validated voter turnout in 2016 among individuals in the nationwide voter file. Here we use the Voter Eligible Population as the denominator for calculating the turnout rate by state. . . . .

S6 Validated Voter Turnout Rates by State, 2014: Mean levels of validated voter turnout in 2014 among individuals in the nationwide voter file. Here we use the Voter Eligible Population as the denominator for calculating the turnout rate by state. . . . .

S7 Validated Voter Turnout Rates by State, 2016: Mean levels of validated voter turnout in 2016 among individuals in the nationwide voter file. Here we use the number of registered voters in the state as the denominator for calculating the turnout rate by state. . . . .

S8 Validated Voter Turnout Rates by State, 2014: Mean levels of validated voter turnout in 2016 among individuals in the nationwide voter file. Here we use the number of registered voters in the state as the denominator for calculating the turnout rate by state. . . . .

S9 Distribution of electoral precinct turnout for all individuals in the nationwide voter files for 2016 and 2014.

S10 Distribution of electoral precinct turnout for Asian individuals compared to White individuals in 2016. As can be seen, Asian people are much more likely to live in lower turnout areas. This can be seen by the leftward shift of the entire turnout distributions for this group compared to Whites. A Kolmogorov-Smirnov test reveals that these distributions are statistically distinct from one another ($p < 0.0001$). . . . .

S11 Distribution of electoral precinct turnout for various groups 2014. As can be seen, minority groups are much more likely to live in lower turnout areas. This is also true of Democrats compared to Repbulicans and young voters compared to older voters. A Kolmogorov-Smirnov test reveals that these distributions are statistically distinct from one another ($p < 0.0001$). . . . .

S12 Distribution of electoral precinct turnout for various groups in 2016 using only states where voter registrations contain the actual race if the voter (AL, FL, GA, LA, NC, SC, MS, TN, as opposed to a modelled race in other states) or the party registration of the voter (AK, AZ, CA, CO, DE, DC, MD, MS, NE, NH, NY, NC, OK, OR, RI, UT, WV, and WY, as opposed to a modelled party in other states). In these states, the differences in precinct turnout persist for Black voters but are smaller for Asian and Latino voters. The difference also persists between Democrats compared to Repbulicans. One consideration is whether these differences are a function of the different way in which race and partisanship are recorded (through voter registration rather than a model) or if they reflect differences across states with different partisan and racial dynamics. . . . .

S13 Proportion of all voters in a precinct living in a turnout desert (i.e. 1 standard deviation below the national average in voter turnout in 2016). . . . .

S14 Proportion of all voters in 2014 in a precinct living in a turnout desert. We define a Turnout Desert as a precinct with 2014 turnout 1 standard deviation below the national average in voter turnout in 2014. . . . .

S15 Proportion of White voters in 2014 in a precinct living in a turnout desert. We define a Turnout Desert as a precinct with 2014 turnout 1 standard deviation below the national average in voter turnout in 2014. . . .

S16 Proportion of Black voters in 2014 in a precinct living in a turnout desert. We define a Turnout Desert as a precinct with 2014 turnout 1 standard deviation below the national average in voter turnout in 2014. . . . .

S17 Proportion of Latino voters in 2014 in a precinct living in a turnout desert. We define a Turnout Desert as a precinct with 2014 turnout 1 standard deviation below the national average in voter turnout in 2014. . . .

S18 Proportion of Asian voters in 2014 in a precinct living in a turnout desert. The top map shows Turnout Deserts in 2016. In the bottom map we define a Turnout Desert as a precinct with 2014 turnout 1 standard deviation below the national average in voter turnout in 2014. . . . .

S19 Proportion of Republican voters in 2014 in a precinct living in a turnout desert. The top map shows Turnout Deserts in 2016. In the bottom map we define a Turnout Desert as a precinct with 2014 turnout 1 standard deviation below the national average in voter turnout in 2014. . . . .

S20 Proportion of Democratic voters in 2014 in a precinct living in a turnout desert. The top map shows Turnout Deserts in 2016. In the bottom map we define a Turnout Desert as a precinct with 2014 turnout 1 standard deviation below the national average in voter turnout in 2014. . . . .

S21 Regression Results Predicting Precinct Turnout Rate . . . .

1 MOTIVATING THE USE OF VOTER FILES TO STUDY TURNOUT DESERTS

To our knowledge, we are the first to employ a dataset as large and as comprehensive as we do to explore the vitally important social patterns in voting at a local level. Our sample is an order of magnitude (or more) larger than previous studies. Some studies use data from individual states' public-use voter registration lists or match surveys to vote files; however, most of these studies examine only one state at a time rather a full nationwide panel of voters or are constrained by the survey design and sample size of the surveys they use. Our data allow us to fully map the dynamics of *validated* voter turnout in the United States with a degree of precision not previously realized. Using these data, we explore the individual- and community-level dynamics of voter turnout in the United States.

As we mentioned in the paper, we use state administrative voter files because our analyses (save for the aggregate nationwide numbers) cannot be run using surveys. Surveys, including very large-n surveys such as the Current Population Survey (CPS) and Cooperative Congressional Election Study (CCES), are simply too small and are not designed to be representative at geographic locations lower than the state level. Only voter files can hope to capture the local dynamics of voting in the United States.

Moreover, *even if we were able to get a large enough survey*, survey measures of voting would still suffer from numerous issues. First among these is over-reporting of voting, which can be a *massive* problem. For example, in the 2018 elections, 88% of citizens surveyed on the CCES said that they voted. However, when the CCES was matched to voter files, only 43% of participants actually voted. This problem is not unique to 2018—in 2016, self-reported voter turnout was 51 percentage points higher than validated voting in the CCES. Somewhat ironically, if researchers prefer the use of surveys over voter files but then use validated vote in those surveys they are in reality using the voter file but on for the small sample of the file that matches with survey respondents. Furthermore, even when surveys match to voter files, there may be large issues with survey's sampling design that make them less valuable than voter files themselves. Indeed, the CCES is quite far off from official turnout numbers among young people at the state level even when looking at validated voting[1]. Furthermore, voting over-reporting is not random—it varies by the crucial dynamics that we explore in the paper. Those who are older, minorities, and Republicans are much more likely to over-report voting[2]. The differences are large and statistically significant.

For these reasons, we cannot use surveys to study turnout deserts. Though surveys are valuable when used appropriately, they have provided a skewed perspective of patterns of turnout in the electorate. For example, the simple bivariate relationship between age and voting, gender and voting, and race and voting is quite different in voter files than in surveys[2]. Regardless of where one stands on the relative merit of survey data, this much is clear: surveys are not the best we can do (and may not even be viable at all given sample size/design issues) to study the role of turnout at the level of small geographic units, like counties, let alone electoral precincts, as we do here.

Without surveys, the best candidate is public use voter files. They are much better situated to answer the questions outlined in this paper and in studies of turnout more generally. Their coverage is an order of magnitude larger than surveys, which allows researchers to look into the dynamics at play in local communities. Furthermore, voter files measure validated vote history, thus taking care of the large, non-random over-reporting of voting.

We note that voter files are not without their issues. Voter files have a certain amount of what industry professionals call "deadwood"—voters who have moved or died but who have not updated their voter records. In addition, some states are more active than others in purging their lists of deadwood and inactive voters. While these patterns do occur in voter files, we have strong reason to believe that they do not influence our results. First, in all of our models we include state fixed effects (and in some cases smaller jurisdictions). This means our statistical identification comes from changes in turnout within states. In all states, state board of elections are the primary arbiters/purgers of voter files. As a result, we are looking within jurisdictions that have constant patterns of voter purging and deadwood. The state fixed

effects, then, address this problem. Even if one believes that smaller jurisdictions are the one's controlling purges of the voter file—after all, counties do handle voter files in their purview—our county and precinct fixed effects would address this concern. Moreover, if one looks at patterns of purging, they appear to be fairly uniform across states[1,3].

Moreover, it is important to note that other potential issues with voter files are relatively small and unlikely to bias estimates of interest. For instance, previous work has noted that the number of potential duplicate observations in the files is incredibly low[3,4]. Further, previous work has shown that modeled variables included in the voter files that we use in the paper (such as race and partisanship) are quite accurate. This is somewhat intuitive given that data vendors (i.e. the political parties and candidates) have a *strong* incentive to get party attachments correct, because their massive voter contact initiatives depend on contacting people of their own party and not of the other. Significant resources are dedicated to modeling race and party. Furthermore, academic research confirms this suspicion by comparing modeled race in voter files to census data and shows a strong correlation[5].

Modeling race consists of comparing names to the racial breakdown of names in the Census along with where an individual lives. This technique creates race predictions that are highly reliable and match very well with race measures from survey self-reports[5]. This is also true of the Pew Foundation's survey panel[6]; they show that modeled race in voter files matches individuals' self-reported race in the survey in more than 8 out of 10 cases. They note, "race and ethnicity are generally well measured in the files"[6]. Moreover, aggregates of race predictions from the voter files align closely with official counts from the Census[5]. Furthermore, if the minimal amount of measurement error in race is random, our results would be attenuated and/or overestimate their levels of uncertainty. If differential measurement error is constant within states, counties, precincts, or individuals themselves, we would account for that with our fixed effects design. Finally, we note that our results hold even if we isolate our analysis to states that report the race that voters included on their voter registration forms. Given the high caliber of modeled race scores, using these has become standard practice in the field[7].

Modeling political party consists of a process that has four steps. The first step takes the political party one is registered with (in places where this is reported). The second step fills in party for those for whom party registration is not available based on whether an individual votes in a specific party's primary. The third step fills in lingering missing individuals based on contact from political parties. The last step uses known individual demographics/location to predict party for whom party has not been identified in steps 1-3. This process creates party scores that are highly reliable. Pew's survey panel matched to several voter file vendors shows that modeled party is strongly related to the party that individuals' self-report[6]—as they note, "modeled partisanship is correct for a majority of cases," with this process matching anywhere between 7-9 in 10 citizens' self-reported party identification. For reference, validated party and self-reported party only match in just over 9 of 10 cases in the CCES, showing that modeled party is fairly close to party registration. Also, aggregates of these party counts to the state or county level align very closely with official election results[1]. Given that partisanship is modeled with a high degree of fidelity, scholars have used information on political leanings in the voter file data to explore a variety of questions; for example, the partisan leanings of physicians, religious leaders, and even spouses[8–10]. Moreover, if the minimal amount of measurement error in party is random, our results would be attenuated and/or overestimate their levels of uncertainty. If differential measurement error is constant within states, counties, precincts, or individuals themselves, we would account for that with our fixed effects models. Finally, we note that our results hold even if we isolate our analysis to the 33 states that report the party that voters included on their voter registration forms.

In short, while no dataset is perfect, voter files are best-suited to study the nature, role, location, and effect of turnout in small geographic units, like electoral precincts, in the United States.

THE ACCURACY OF VOTER FILE MEASURES

We provide three checks of the Data Trust's data quality, similar to what others have shown in previous research[1, 11]. Whatever validation technique is used, however, the information in the voter files about race, age, party, and the number of individual all together—modeled or directly reported—are of high quality and reliability.

Our first check is to simply compare the number of individuals in the voter file and the number of voting age adults in the state (from the Census). We would not expect these two numbers to be exactly the same as not all adults register to vote. However, this check can help us see whether voter files have large amounts of "deadwood", or on the flip side, excess purging.

Figure S1 plots the number of registered voters available in each state. The graph is sorted by the number of citizens registered to vote (dark bars), with the number of voting age population estimates also shown as a benchmark. As can be seen, the number of registered voters at the state level correlates strongly with estimates of the number of people of voting age in the state as a whole ($R = 0.98$). This strong correlation with the voting age population suggests that voter files are not missing large swaths of the registered population across states and are not also including large pools of "deadwood". This is consistent with findings that the number of votes cast in an election is very close to counts reported by Michael McDonald's well-respected and widely used official counts from the U.S. Elections Project[1, 3].

Figure S1. Data Trust Registration Counts and VAP by State

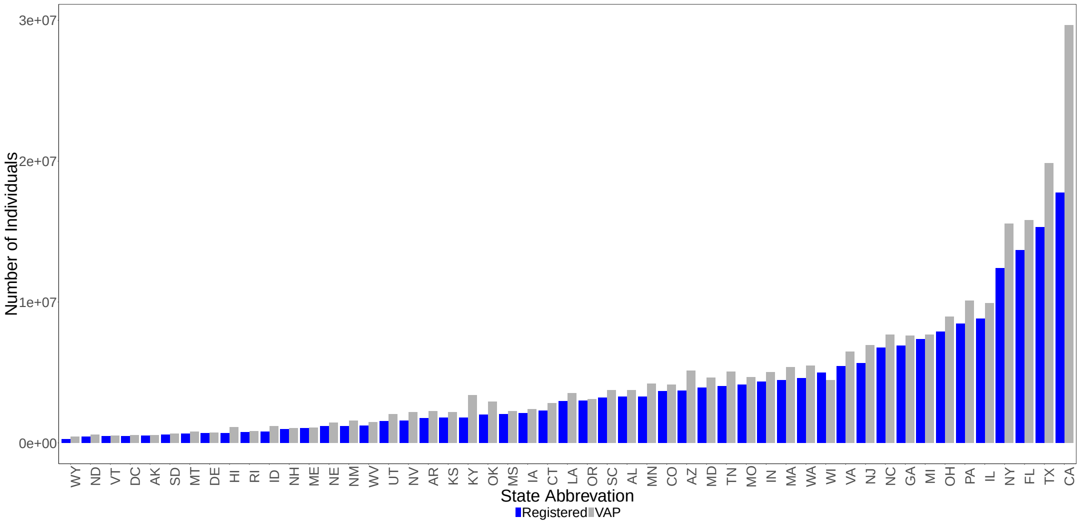

Comparison between number of registered voters in each state from the voter file (dark bars) and the number of voting age individuals by state from the US Census (light bars).

Second, when it comes to racial estimates, the voter files estimate the percent of White registered voters at around 63% and other racial groups comprising the rest of the population. In its estimate closest to the voter file snapshot date (early 2017), the Census estimated that among the entire population 60.7% of is white.[1] For reference, the respective census numbers for African Americans is 13.1% vs. 11.4% in the voter file. While this comparison is not exact, it suggests that the topline estimates are close to one another. Part of the reason for having a slightly higher percentage of whites in the file may be due to racial patterns in voter registration since the voter file is only capturing registered adults while the census is measuring all adults of voting age, registered and unregistered. In the voter files, a clear age gradient in registration can be seen, as has been shown in previous (mostly survey-based) research on voting.

[1] See "Census Quick Facts," *United States Census Bureau*, 7/1/2017

Digging into this further, as can be seen in Figure S2, this approach to coding race benchmarks well with official reports at the state and county level. While not perfect (as would be case if all points where on the 45 degree reference line), the relationship is still very strong regardless of whether we look at the state or county level (State $R = 0.94$; County $R = 0.97$).

Third, when it comes to modeled political party, the Data Trust estimates that about 29% of individuals in its sample are Republicans, 34% are Democrats, and 37% are Independents/Others. The estimates are fairly accurate compared to survey data; for reference, Gallup's running series estimated that among the entire population those numbers are 29%, 30%, and 40% respectively.[2] The voter files seems to get very close to the Republican number and slightly overestimates the number of Democrats relative to Independents in the population. Some of these differences might simply be because citizens who register are different from the population as a whole that Gallup is trying to measure/approximate.

Figure S2. Data Trust Black Variable and Census Black Population Counts

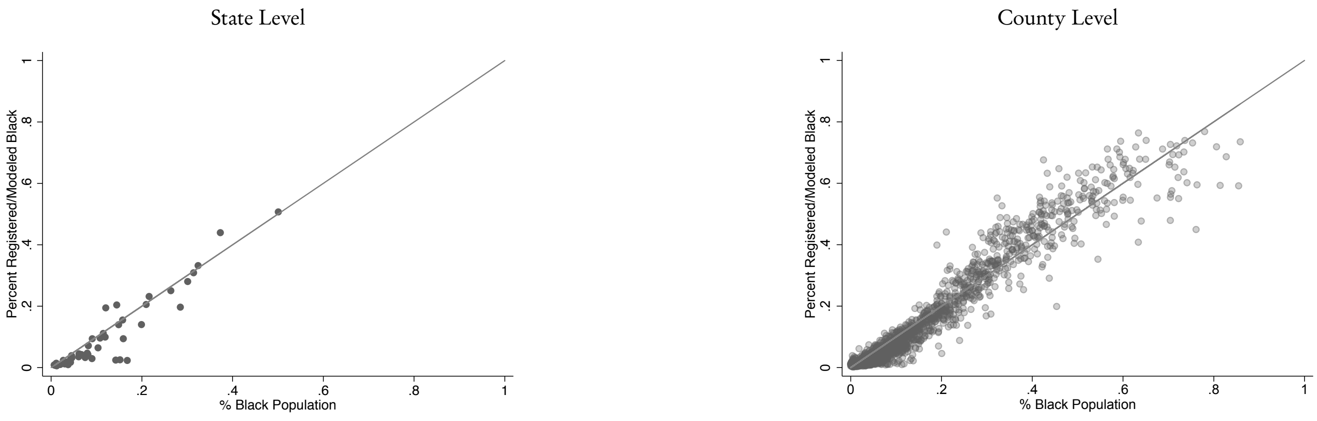

Relationship between the percent of the population that is African American (from the Census) and the Data Trust's race variable, which is a hybrid between registered race (where it is available) and modeled raced based on name and geography. It plots the relationship at two levels: state (left) and county (right). Pearson's R: 0.94 (left), 0.97 (right).

In addition, two other factors suggest that modeled party is of high quality. First, linking Pew survey data to commercial voter file vendors shows that party estimates at the individual-level are quite accurate[6], *even in* states where party registration is not available in the voter files. Second, we can benchmark the voter file's party measures/estimates with presidential vote returns at the state level. As can be seen in Figure S3, as best we can tell, the voter file's modeled party estimates stack up well with official returns. The correlation between modeled party and presidential vote share and modeled party is 0.82. The Data Trust appears to slightly underestimate the proportion of people voting Republican in the file in 2016; however, this appears to be fairly systematic and uniform across states. Furthermore, we would expect some slippage as partisan registration is not a perfect predictor of vote choice in any given election, particularly in 2016 when Donald Trump was the Republican nominee.

[2]See "Party Affiliation," *Gallup* September 6-10 measurement.

Figure S3. Data Trust Modeled Party Relative to Presidential Vote Share (2016)

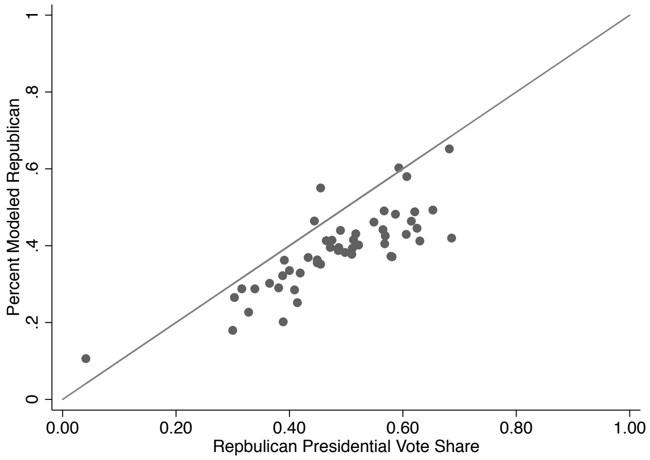

Relationship between Republican vote share (from official counts) and the Data Trust's modeled party score. Pearson's R: 0.82.

TURNOUT RATES NATIONWIDE IN 2016 AND 2014

Figure S4 shows the mean levels of voter turnout among individuals in the nationwide voter file by the two years that we explore. The first bar on the left of both panels shows the rate among everyone in the dataset. The rest of the bars show turnout rates among various subgroups. To calculate voting rates we use the number of individuals who voted divided by the number of registered voters in the file. In the main paper we calculate the voting rate using the number of individuals who voted divided by the number of people in the voting eligible population (VEP) as calculated by the US Census Bureau. The turnout rates between Figures S4 and the main paper increase because the VEP includes unregistered individuals. However, the gaps across groups within each figure persist.

Figure S4. Gaps in Validated Voter Turnout Rates by Groups using Voter File as Denominator

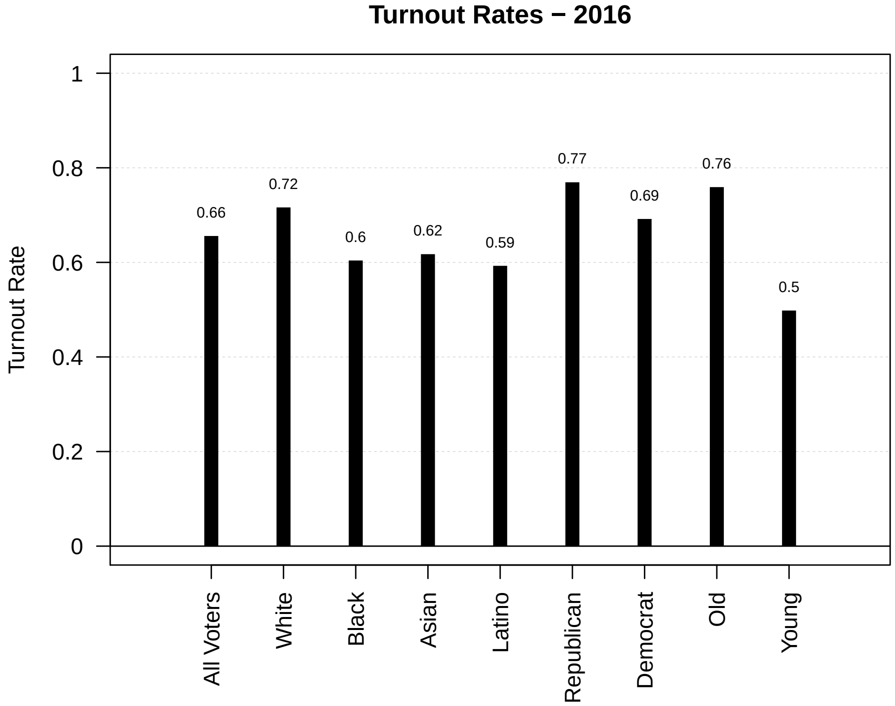

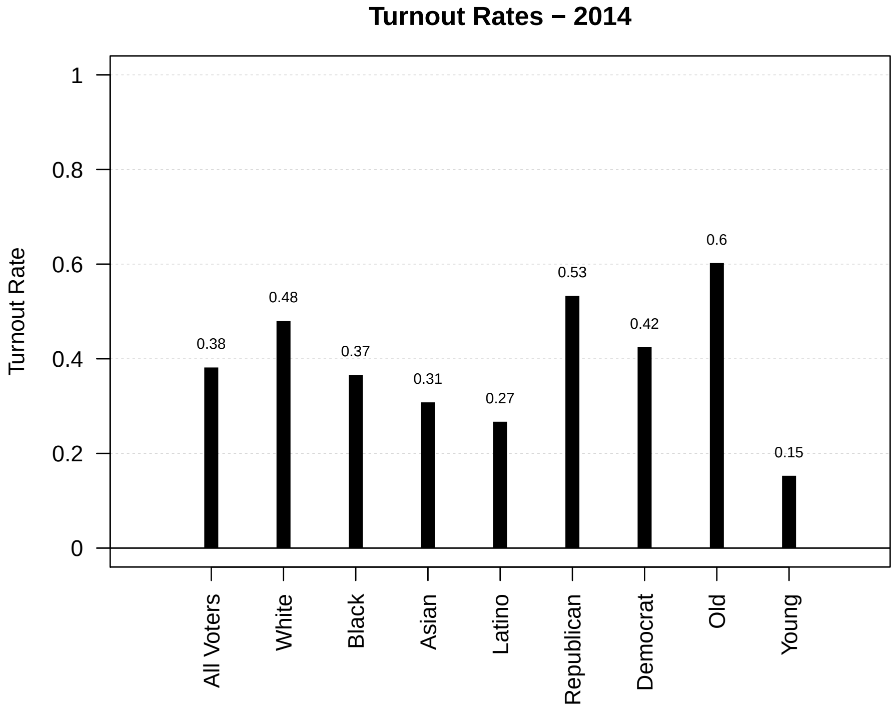

Mean levels of voter turnout in 2016 (top) and 2014 (bottom) among individuals in the nationwide voter file. We use the number of registered voters from the nationwide voter file for the denominator to determine voting rates.

TURNOUT RATES FOR INDIVIDUAL STATES IN 2016 AND 2014

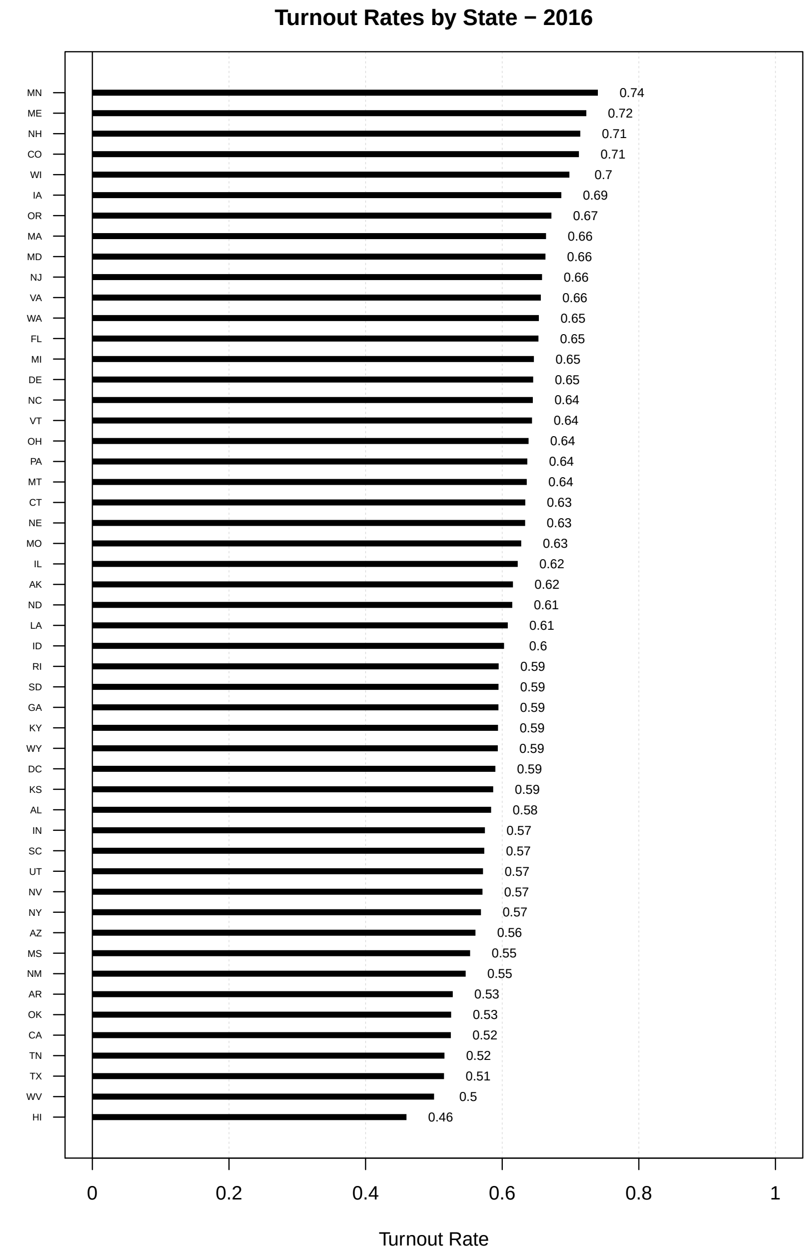

Figure S5. Validated Voter Turnout Rates by State, 2016: Mean levels of validated voter turnout in 2016 among individuals in the nationwide voter file. Here we use the Voter Eligible Population as the denominator for calculating the turnout rate by state.

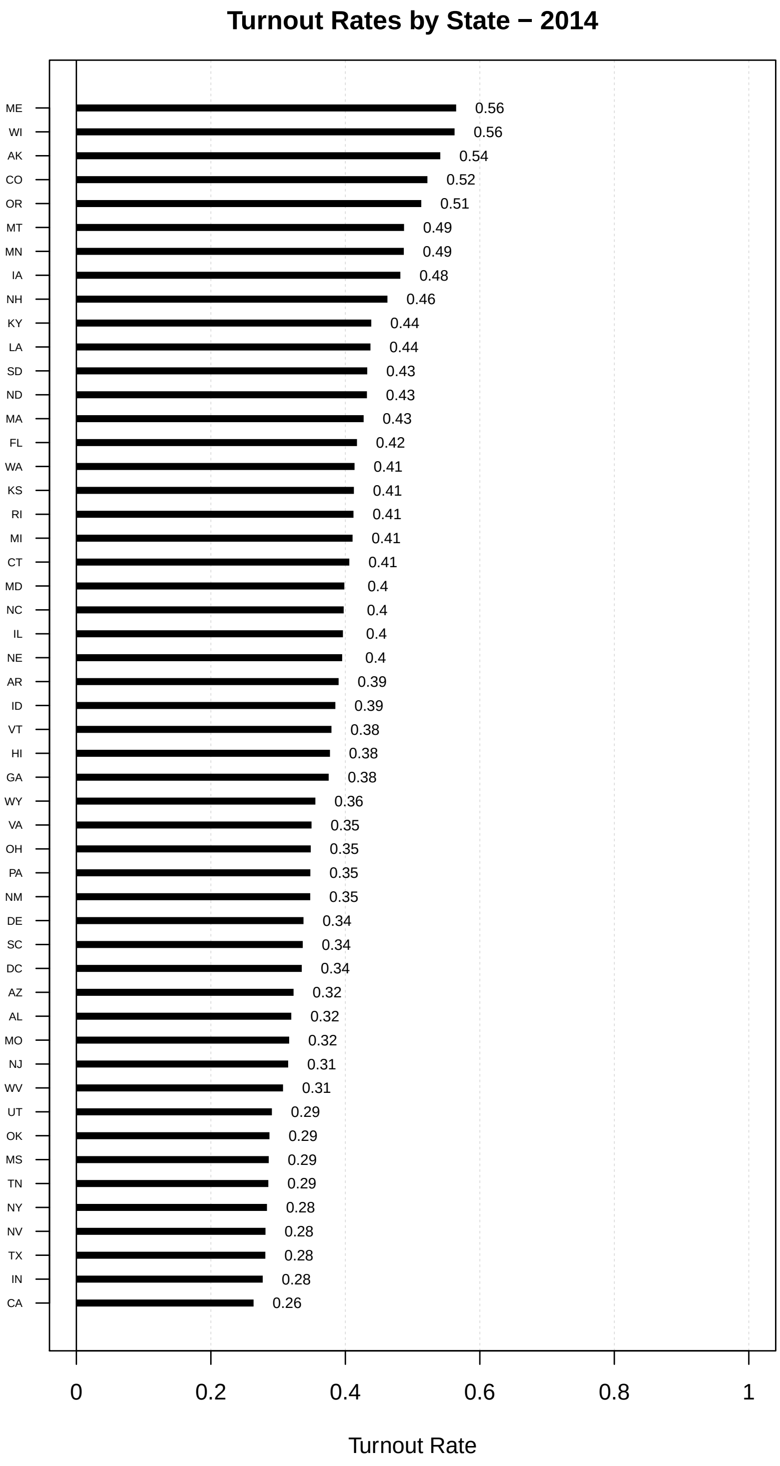

Figure S6. Validated Voter Turnout Rates by State, 2014: Mean levels of validated voter turnout in 2014 among individuals in the nationwide voter file. Here we use the Voter Eligible Population as the denominator for calculating the turnout rate by state.

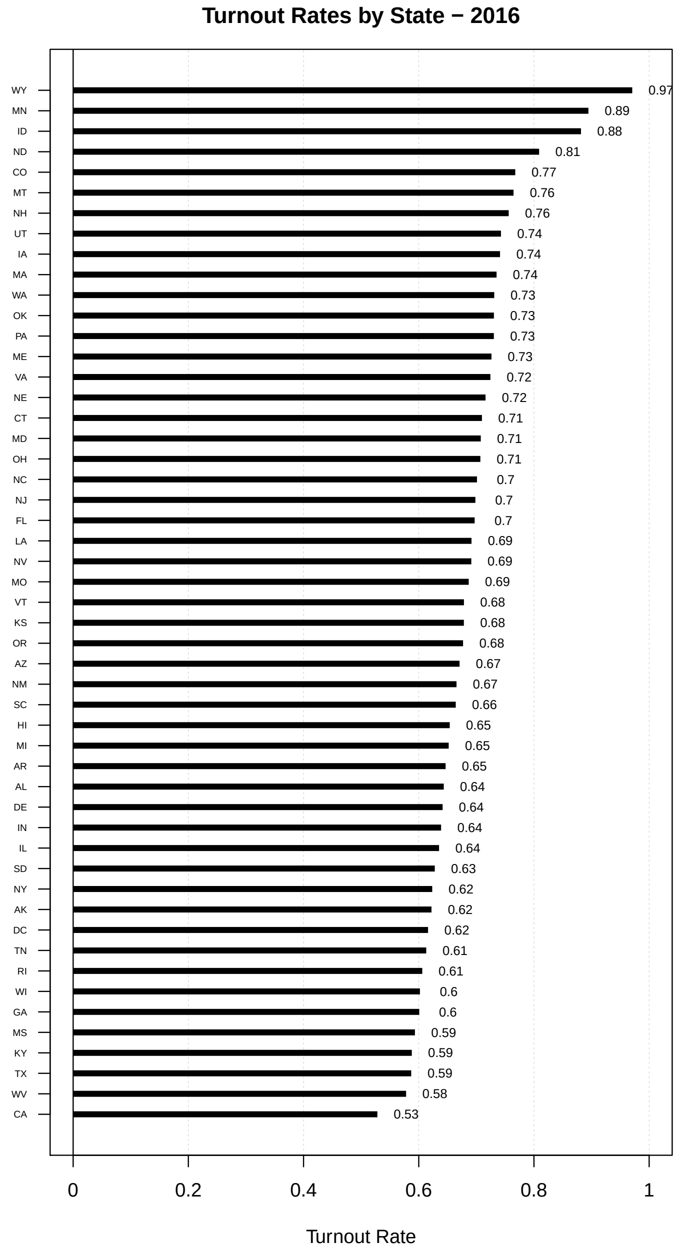

Figure S7. Validated Voter Turnout Rates by State, 2016: Mean levels of validated voter turnout in 2016 among individuals in the nationwide voter file. Here we use the number of registered voters in the state as the denominator for calculating the turnout rate by state.

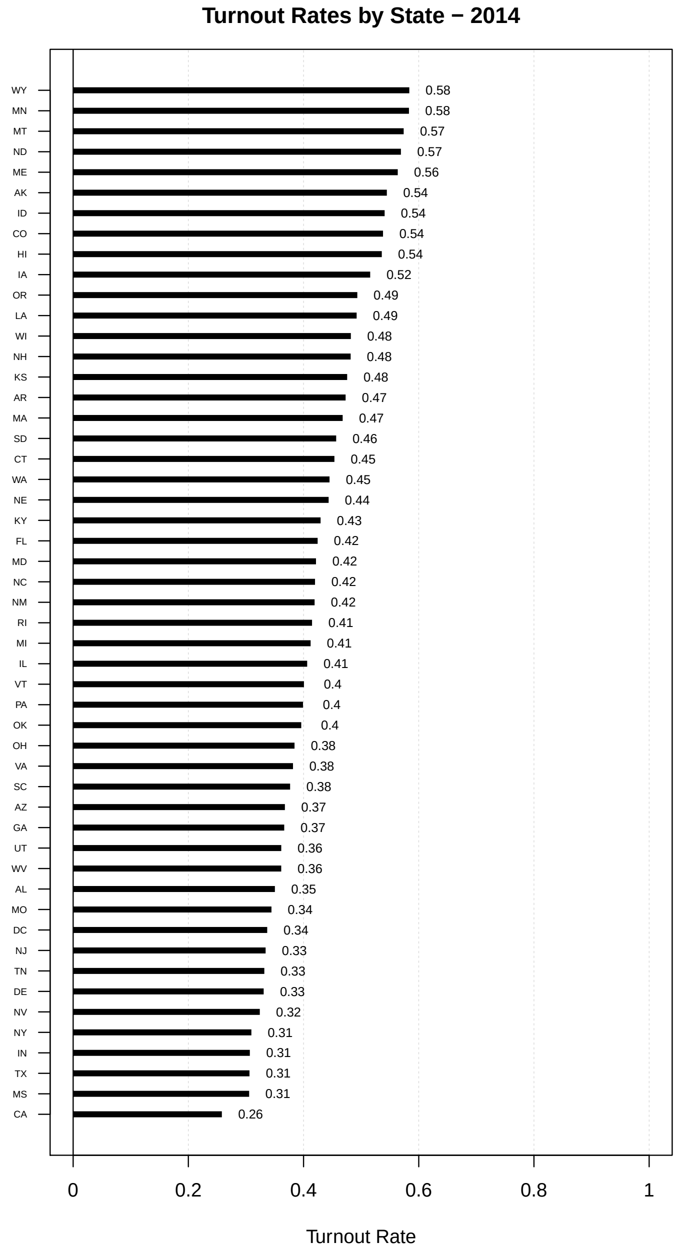

Figure S8. Validated Voter Turnout Rates by State, 2014: Mean levels of validated voter turnout in 2016 among individuals in the nationwide voter file. Here we use the number of registered voters in the state as the denominator for calculating the turnout rate by state.

ADDITIONAL DISTRIBUTIONS OF PRECINCT TURNOUT RATES

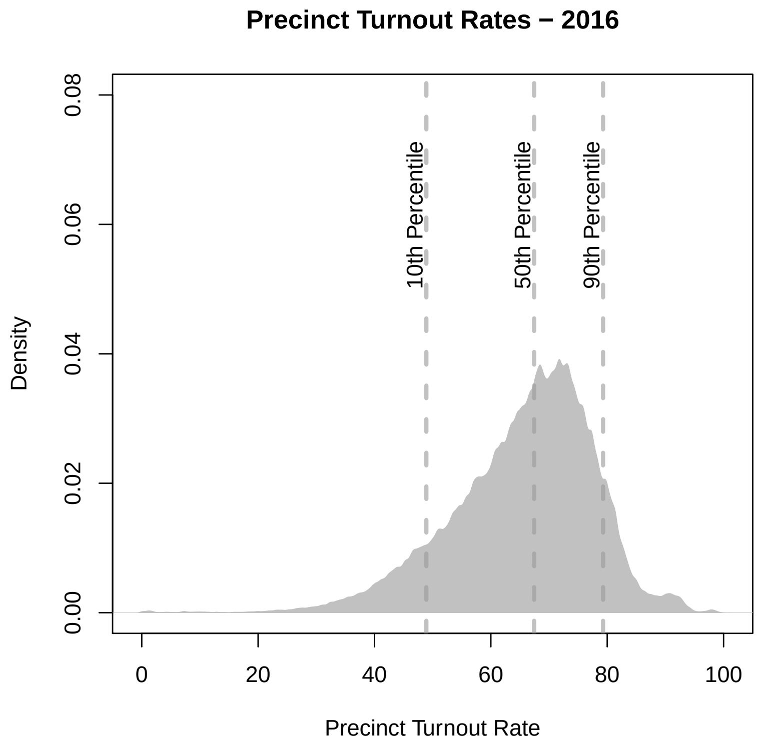

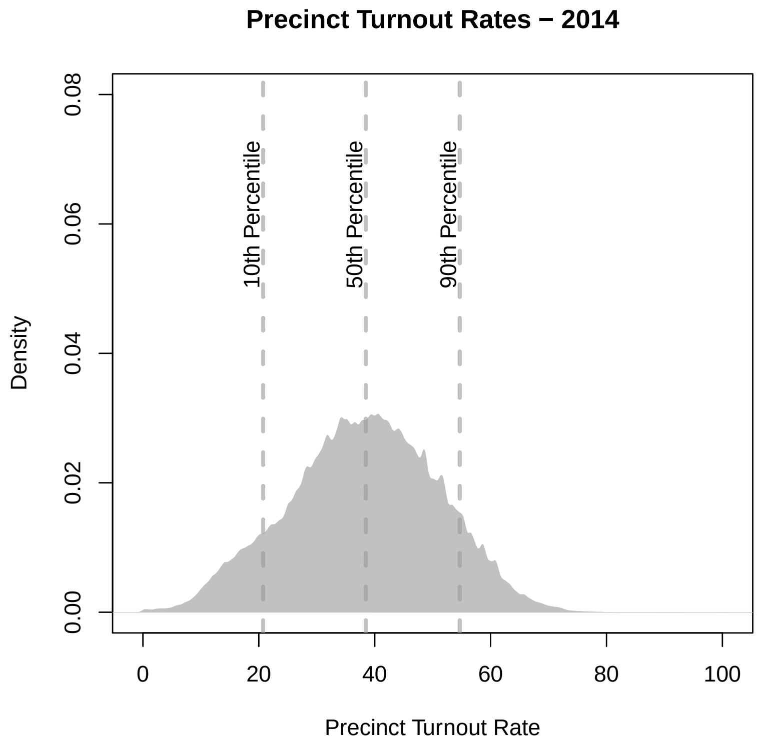

Figure S9. Distribution of electoral precinct turnout for all individuals in the nationwide voter files for 2016 and 2014.

Our results hold if we only look at states where party or race information comes from registration forms. In race reg-

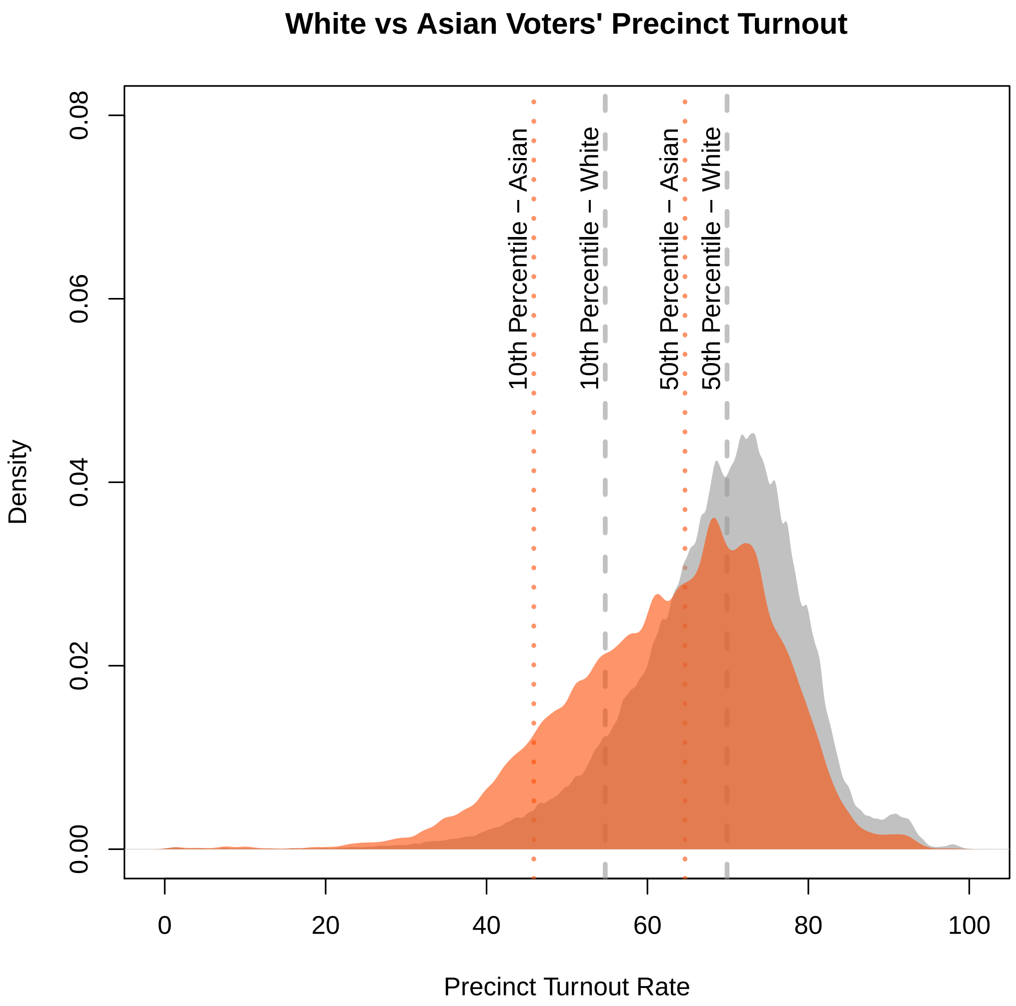

Figure S10. Distribution of electoral precinct turnout for Asian individuals compared to White individuals in 2016. As can be seen, Asian people are much more likely to live in lower turnout areas. This can be seen by the leftward shift of the entire turnout distributions for this group compared to Whites. A Kolmogorov-Smirnov test reveals that these distributions are statistically distinct from one another ($p < 0.0001$).

istration states, on average Asian people live in precincts with turnout rates 1.3 percentage points lower than whites, with that number being 4.7 percentage points for Black people, 4.8 percentage points for Latinx people, and 1.5 percentage points for people of other races. For party registration states, Democrats on average live in precincts where turnout is 2.62 percentage points lower.

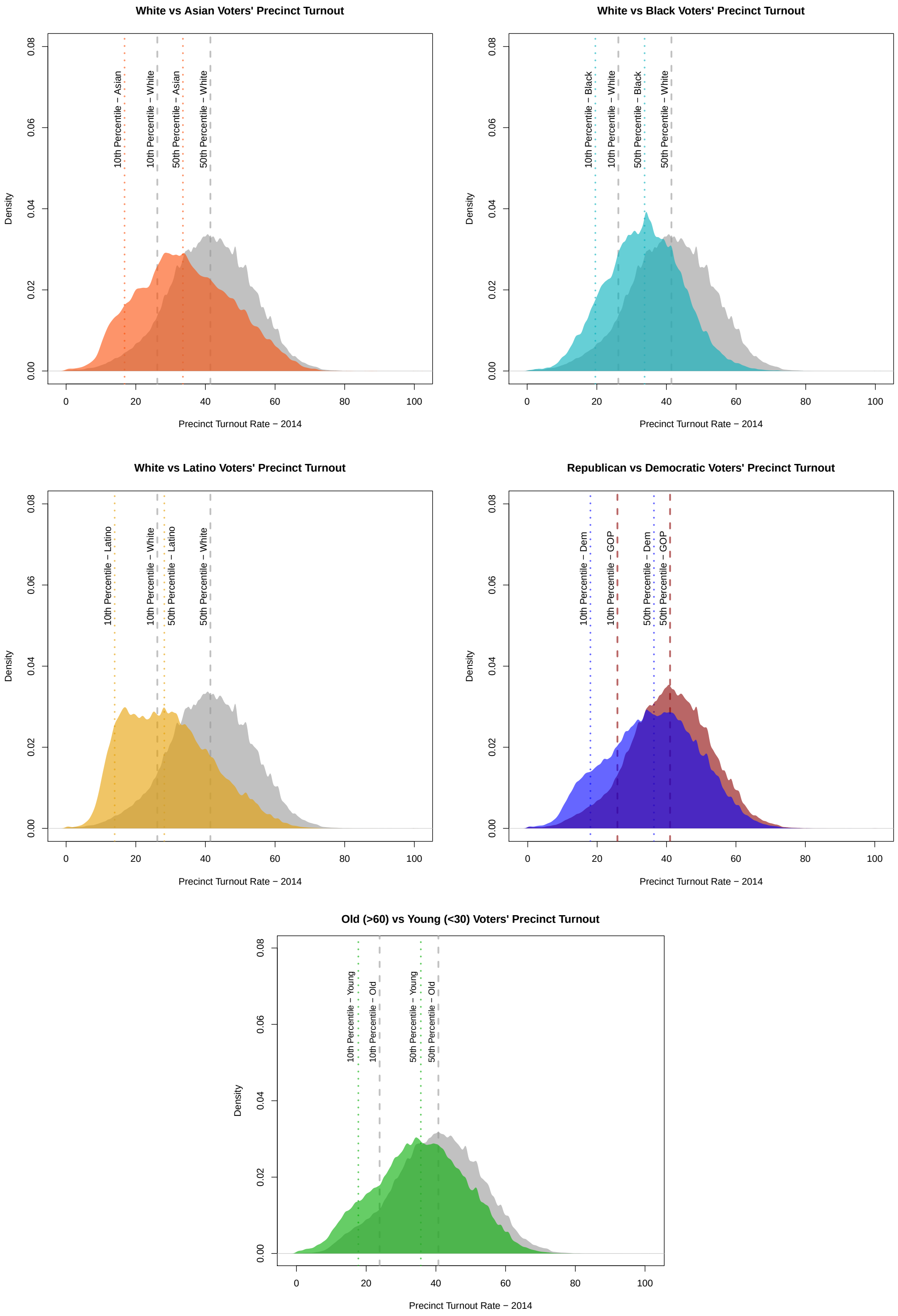

Figure S11. Distribution of electoral precinct turnout for various groups 2014. As can be seen, minority groups are much more likely to live in lower turnout areas. This is also true of Democrats compared to Repbulicans and young voters compared to older voters. A Kolmogorov-Smirnov test reveals that these distributions are statistically distinct from one another ($p < 0.0001$).

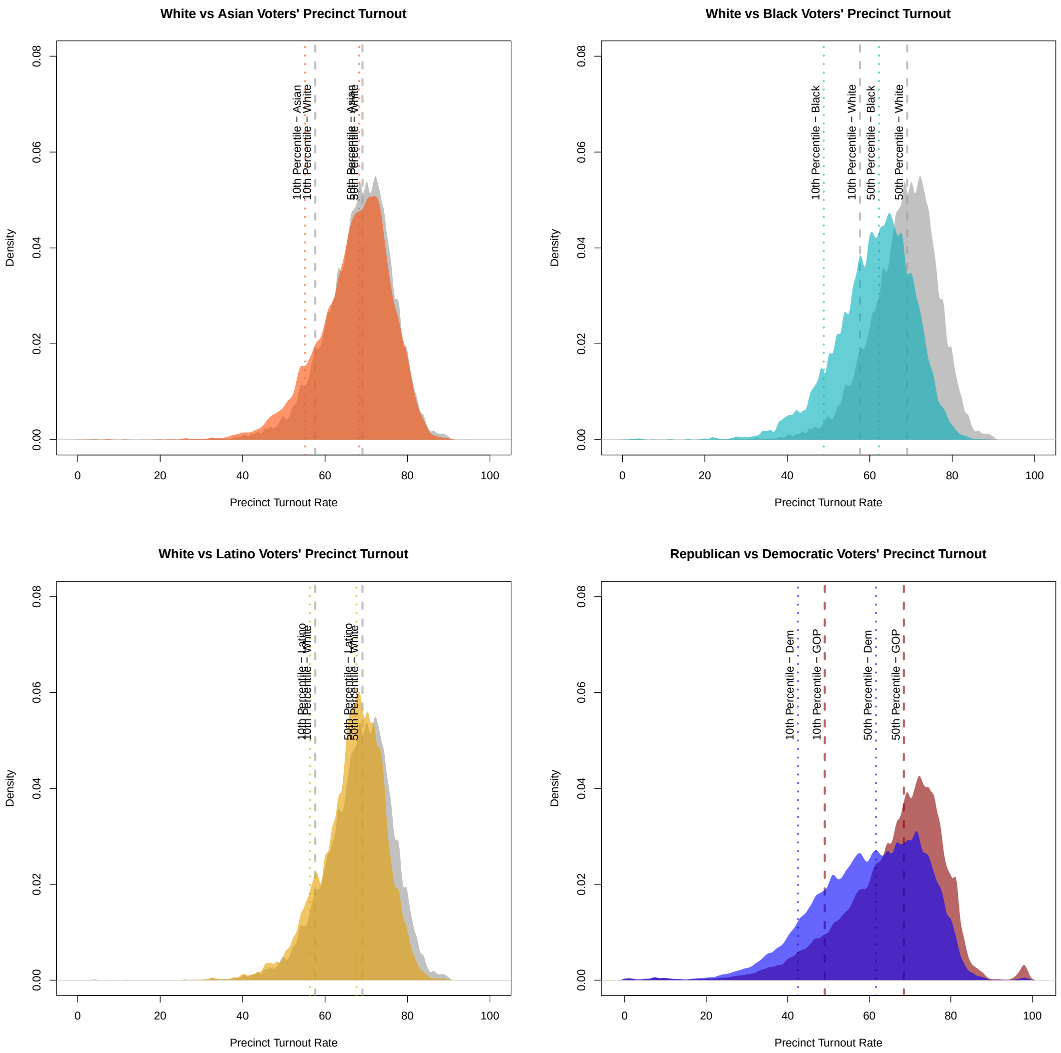

Figure S12. Distribution of electoral precinct turnout for various groups in 2016 using only states where voter registrations contain the actual race if the voter (AL, FL, GA, LA, NC, SC, MS, TN, as opposed to a modelled race in other states) or the party registration of the voter (AK, AZ, CA, CO, DE, DC, MD, MS, NE, NH, NY, NC, OK, OR, RI, UT, WV, and WY, as opposed to a modelled party in other states). In these states, the differences in precinct turnout persist for Black voters but are smaller for Asian and Latino voters. The difference also persists between Democrats compared to Repbulicans. One consideration is whether these differences are a function of the different way in which race and partisanship are recorded (through voter registration rather than a model) or if they reflect differences across states with different partisan and racial dynamics.

MAPS OF TURNOUT DESERTS

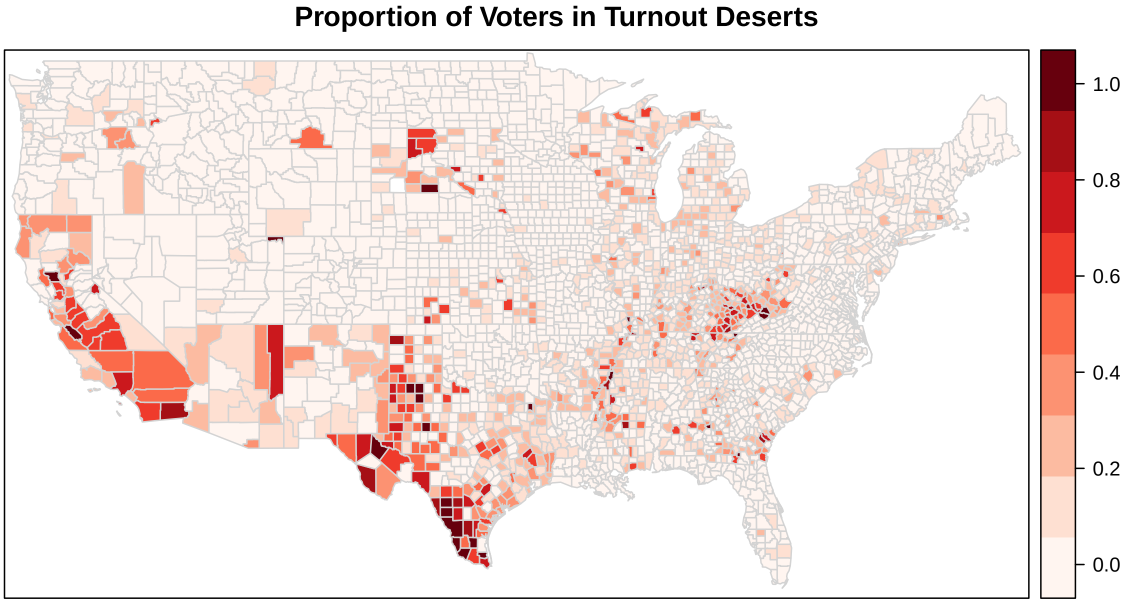

Figure S13. Proportion of all voters in a precinct living in a turnout desert (i.e. 1 standard deviation below the national average in voter turnout in 2016).

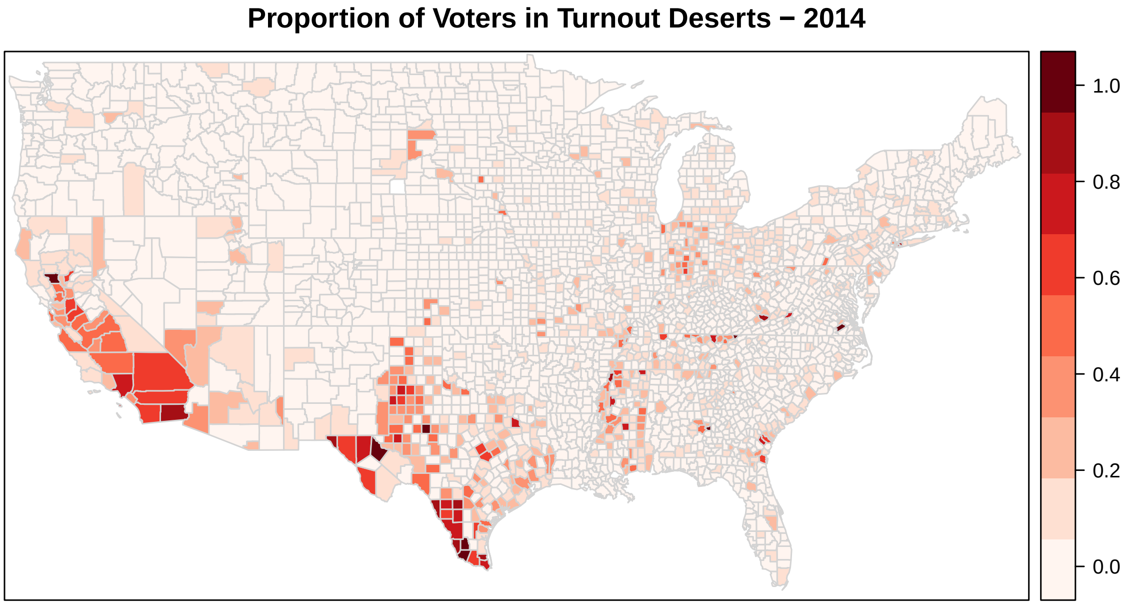

Figure S14. Proportion of all voters in 2014 in a precinct living in a turnout desert. We define a Turnout Desert as a precinct with 2014 turnout 1 standard deviation below the national average in voter turnout in 2014.

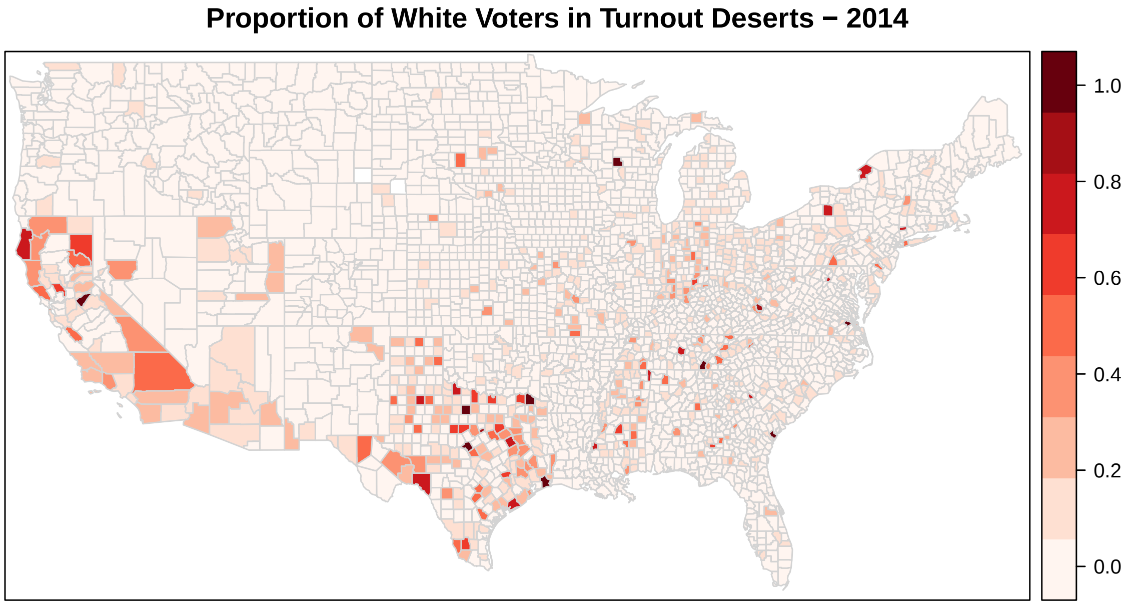

Figure S15. Proportion of White voters in 2014 in a precinct living in a turnout desert. We define a Turnout Desert as a precinct with 2014 turnout 1 standard deviation below the national average in voter turnout in 2014.

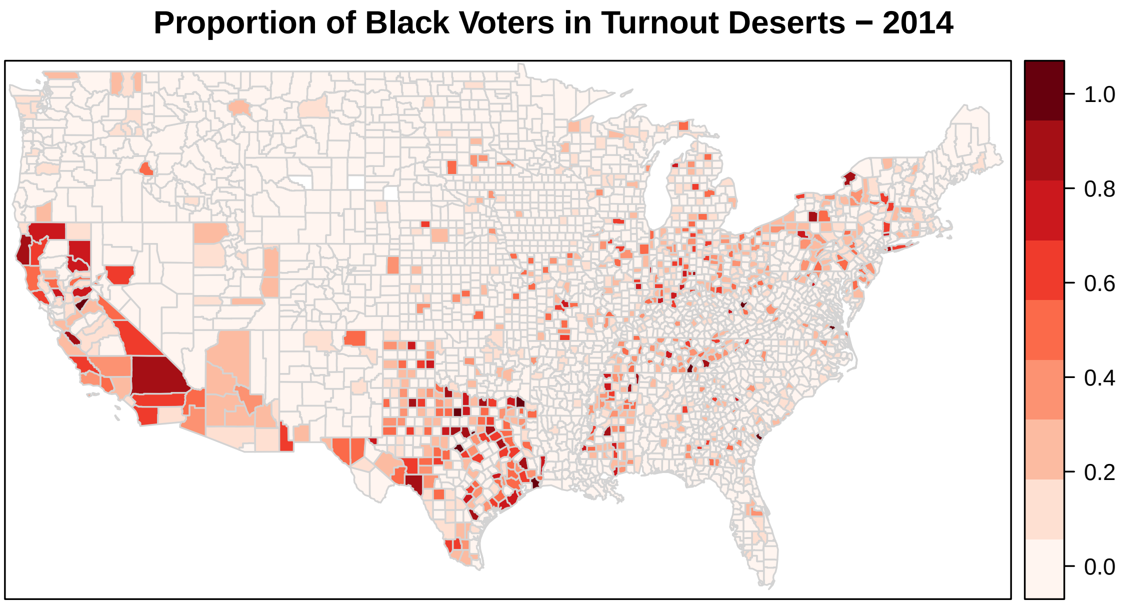

Figure S16. Proportion of Black voters in 2014 in a precinct living in a turnout desert. We define a Turnout Desert as a precinct with 2014 turnout 1 standard deviation below the national average in voter turnout in 2014.

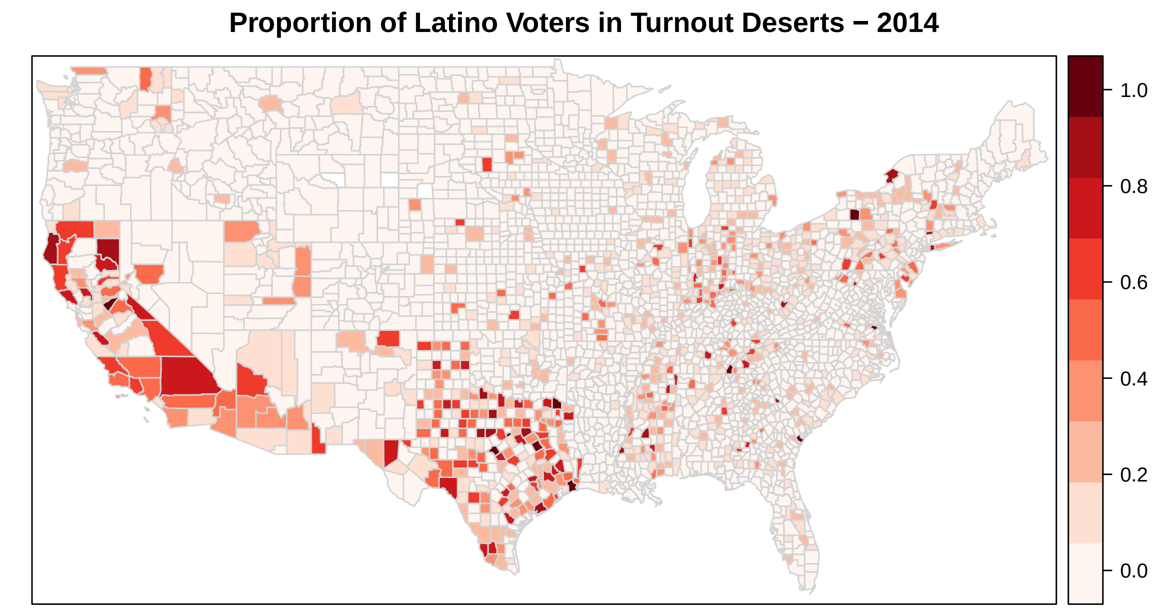

Figure S17. Proportion of Latino voters in 2014 in a precinct living in a turnout desert. We define a Turnout Desert as a precinct with 2014 turnout 1 standard deviation below the national average in voter turnout in 2014.

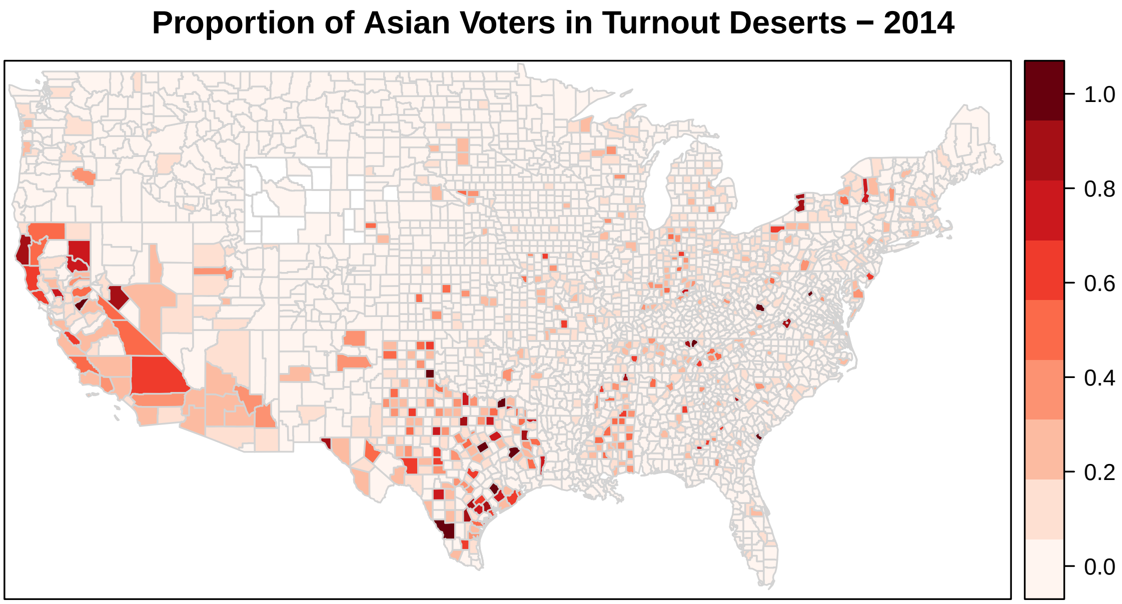

Figure S18. Proportion of Asian voters in 2014 in a precinct living in a turnout desert. The top map shows Turnout Deserts in 2016. In the bottom map we define a Turnout Desert as a precinct with 2014 turnout 1 standard deviation below the national average in voter turnout in 2014.

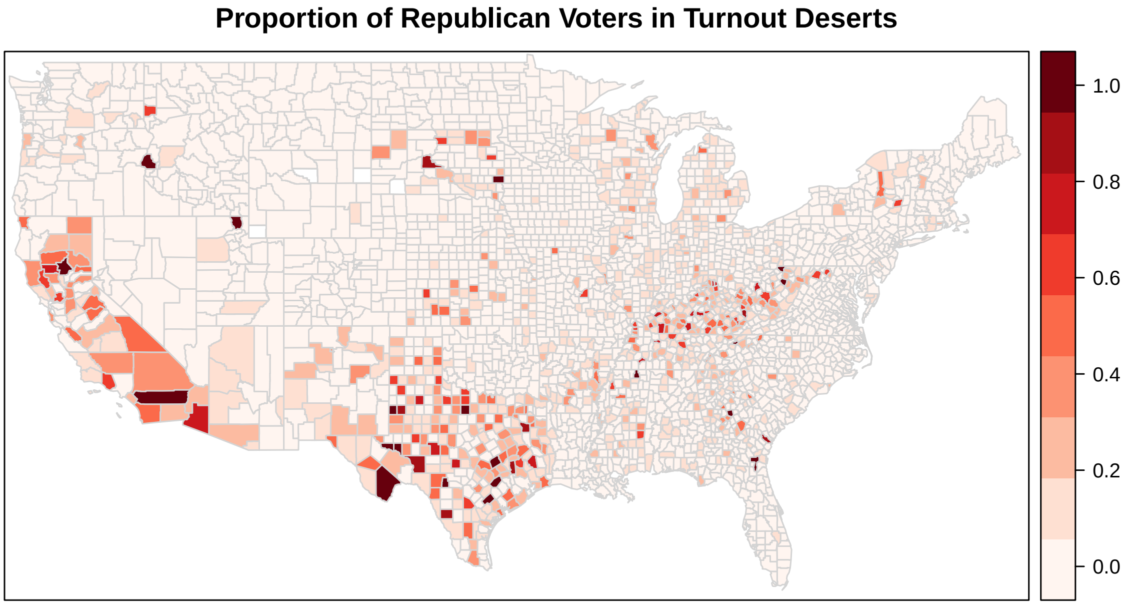

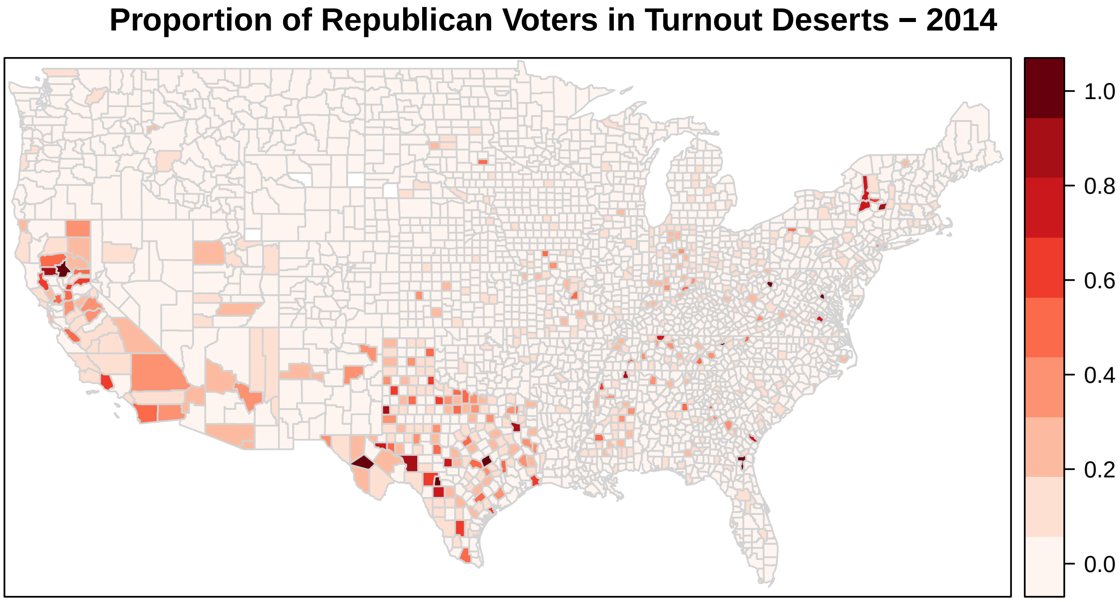

Figure S19. Proportion of Republican voters in 2014 in a precinct living in a turnout desert. The top map shows Turnout Deserts in 2016. In the bottom map we define a Turnout Desert as a precinct with 2014 turnout 1 standard deviation below the national average in voter turnout in 2014.

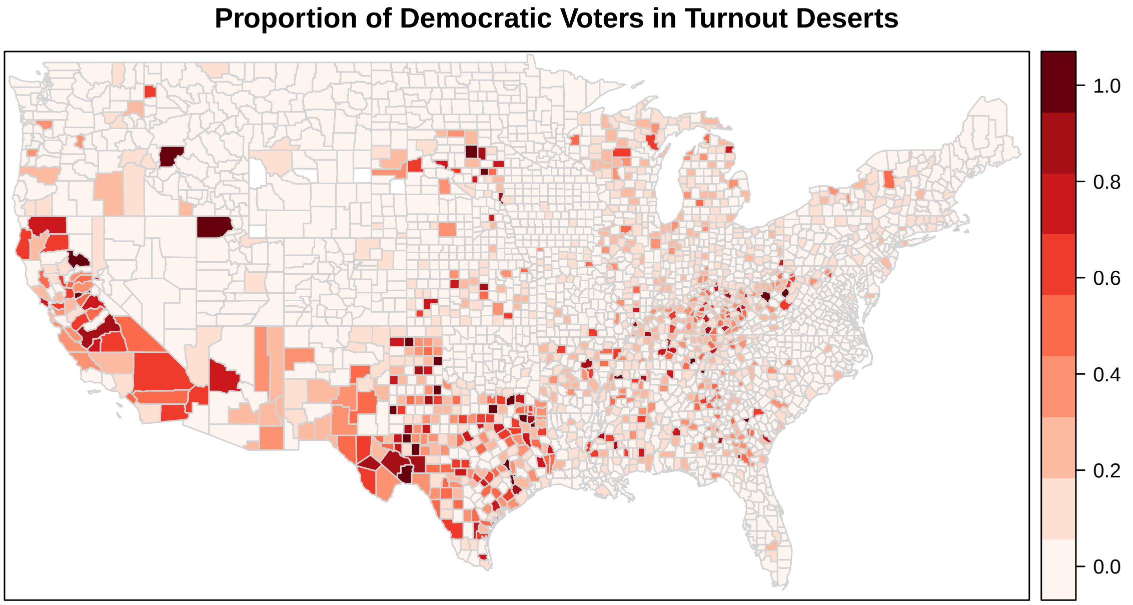

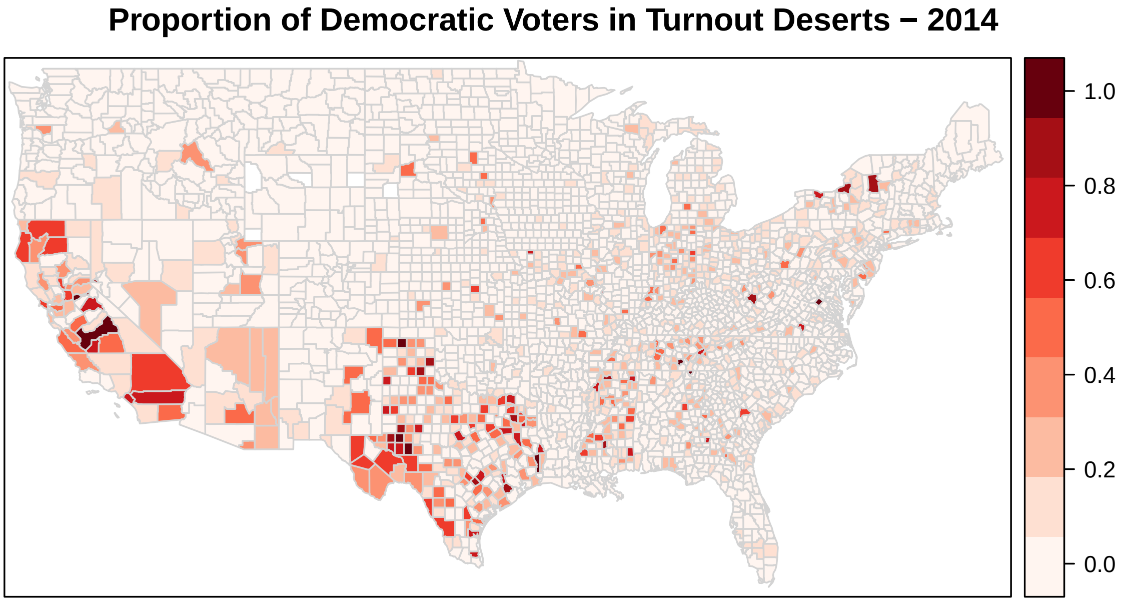

Figure S20. Proportion of Democratic voters in 2014 in a precinct living in a turnout desert. The top map shows Turnout Deserts in 2016. In the bottom map we define a Turnout Desert as a precinct with 2014 turnout 1 standard deviation below the national average in voter turnout in 2014.

1.1 *Additional Regression Results*

To account for the correlations between race, gender, partisanship and age, we conduct a multiple regression model in which the dependent variable is the turnout rate of each voter's precinct. We then include each of these demographic variables in the regression model to estimate the independent relationship between the demographic features we have shown above and living in an area with higher or lower turnout. We estimate a separate model for 2014 and 2016 and include state fixed effects in each model. Standard errors are clustered by precinct.

Figure 5 in the main paper displays the results of these regression models. Here we include additional variables measuring the proportion of a voter's precinct by racial category and partisanship. When we do so, the size of the coefficients for many of the individual demographic factors, particularly race, are diminished.

This presents an interesting result given that in the original models the coefficients on race were largely negative and substantively larger. This suggests that the results that suggest minorities tend to live in areas with low turnout is largely due to the racial composition of the precinct. In other words, the negative coefficients on the % precinct race variables indicates that precincts in which a larger number of minorities live tend to also tend to have lower turnout. And once we account for the precinct's racial composition, the probability that an individual voter of that race lives in a precinct with low turnout is near zero, or if anything, slightly positive. In other words, minorities are more likely to live in lower turnout precincts (the result from the original models) largely because minority voters are less likely to turn out to vote and people of the same ethnicity are more likely to live near one another, which in the aggregate creates precincts with lower turnout.

For example, the coefficient on the variable 'precinct % Black' is -0.16. This suggests that an increase in 50 percentage points in a precinct's Black residents is associated with an overall decline in precinct turnout rate of 8 percentage points (-0.16 x 50 = -0.8), roughly equal to the coefficient on Black in the regression models in the main paper that did not account for the racial composition of the precinct.

Figure S21. Regression Results Predicting Precinct Turnout Rate

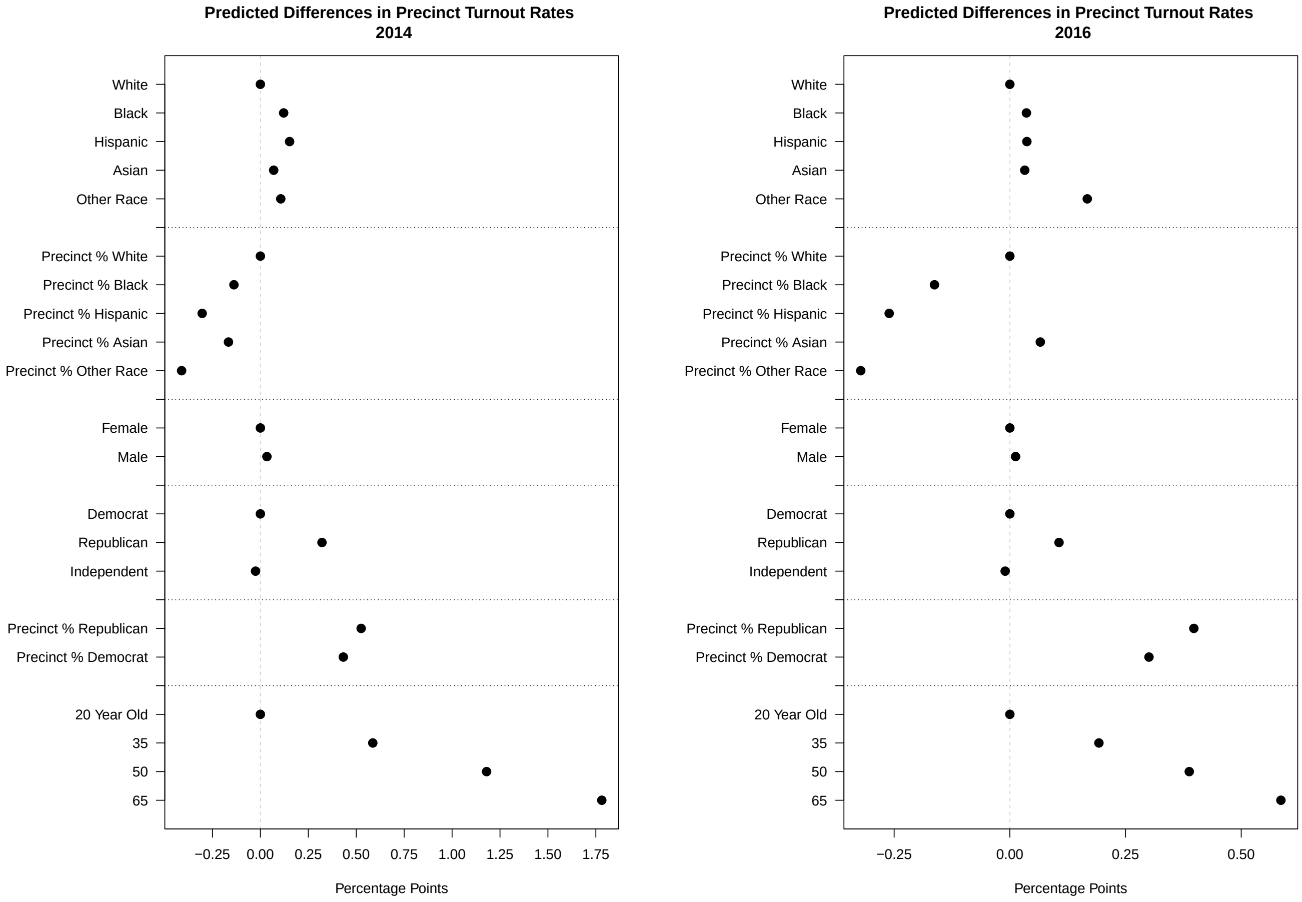

Note: Each figure shows coefficients from a model where a voter's precinct turnout rate is the dependent variable. Coefficients show predicted differences in turnout rates for various demographic characteristics of voters. Each model includes state fixed effects and standard errors are clustered by precinct. Confidence intervals are not shown because the very large sample size (150-200 million in each model) leads to intervals that are incredibly small. Precinct race and partisan composition varibles are measured on a 0-100 scale.

REFERENCES

1. B. Fraga, J. Holbein, *Electoral Studies* p. 102086 (2019).

2. S. Ansolabehere, E. D. Hersh, *Political Analysis* **20**, 437 (2012).

3. B. L. Fraga, J. B. Holbein, C. Skovron, *Working Paper* .

4. S. Goel, M. Meredith, M. Morse, D. Rothschild, H. Shirani-Mehr, *American Political Science Review* (2020).

5. K. Imai, K. Khanna, *Political Analysis* **24**, 263 (2016).

6. R. Igielnik, S. Keeter, C. Kennedy, B. Spahn, *Pew Research Center Report, Washington, DC, available at www. pewresearch. org/2018/02/15/commercial-voter-files-and-the-study-of-us-politics* (2018).

7. B. L. Fraga, *The turnout gap: Race, ethnicity, and political inequality in a diversifying America* (Cambridge University Press, 2018).

8. E. D. Hersh, M. N. Goldenberg, *Proceedings of the National Academy of Sciences* **113**, 11811 (2016).

9. E. D. Hersh, G. Malina, Partisan pastor: the politics of 130,000 american religious leaders, *Tech. rep.*, Working Paper (2017).

10. E. Hersh, Y. Ghitza, *PloS one* **13** (2018).

11. S. Ansolabehere, E. Hersh, *APSA 2011 Annual Meeting Paper* (2011).
